# Digitoids: a novel computational platform for mimicking oxygen-dependent firing of neurons *in vitro*

**DOI:** 10.3389/fninf.2025.1549916

**Published:** 2025-07-01

**Authors:** Rachele Fabbri, Ermes Botte, Arti Ahluwalia, Chiara Magliaro

**Affiliations:** ^1^Research Center “E. Piaggio”, University of Pisa, Pisa, Italy; ^2^Department of Information Engineering (DII), University of Pisa, Pisa, Italy; ^3^Interuniversity Center for the Promotion of 3R Principles in Teaching and Research (Centro 3R), Pisa, Italy

**Keywords:** *in silico* modeling, neuron firing, oxygen metabolism, *in vitro* neuronal network, digitalized neuronal network

## Abstract

**Introduction:**

Computational models are valuable tools for understanding and studying a wide range of characteristics and mechanisms of the brain. Furthermore, they can also be exploited to explore biological neural networks from neuronal cultures. However, few of the current in silico approaches consider the energetic demand of neurons to sustain their electrophysiological functions, specifically their well-known oxygen-dependent firing.

**Methods:**

In this work, we introduce Digitoids, a computational platform which integrates a Hodgkin-Huxley-like model to describe the time-dependent oscillations of the neuronal membrane potential with oxygen dynamics in the culture environment. In Digitoids, neurons are connected to each other according to Small-World topologies observed in cell cultures, and oxygen consumption by cells is modeled as limited by diffusion through the culture medium. The oxygen consumed is used to fuel their basal metabolism and the activity of Na^+^-K^+^-ATP membrane pumps, thus it modulates neuronal firing.

**Results:**

Our simulations show that the characteristics of neuronal firing predicted throughout the network are related to oxygen availability. In addition, the average firing rate predicted by Digitoids is statistically similar to that measured in neuronal networks *in vitro*, further proving the relevance of this platform.

**Dicussion:**

Digitoids paves the way for a new generation of *in silico* models of neuronal networks, establishing the oxygen dependence of electrophysiological dynamics as a fundamental requirement to improve their physiological relevance.

## 1 Introduction

Exploring how neurons process and transmit information is crucial for advancing our knowledge of the brain. Along with the study of biological neural networks in cultures or in *in vitro* slices (Chiappalone et al., 2019; Compte et al., 2003; Humpel, 2015; Van Pelt et al., 2005), computational, or *in silico*, models have been successfully exploited, e.g., to support the study of neuronal network modulation and delineate potential mechanisms underlying activity patterns (Doorn et al., 2023; Masquelier and Deco, 2013; Sukenik et al., 2021; Wen et al., 2022). Several model-based solutions for generating virtual representations of neural cells able to replicate the salient properties of experimentally observed behaviors have been proposed (Lonardoni et al., 2015). For instance, intuitive and easy to use simulators (e.g., BRIAN 2, NEST, NEURON) have been employed to simulate spiking neural network models (Gewaltig and Diesmann, 2007; Hines and Carnevale, 2001; Stimberg et al., 2019). Traditionally, they include mathematical descriptions of the single-neuron activity, ranging from simple phenomenological characterization of neuronal spiking (Izhikevich, 2003) to more complex, biophysical conductance-based simulations of ion fluxes between the intra and the extracellular space (Hodgkin and Huxley, 1952b), as well as models of cell-cell connections to replicate the neuronal network architecture (Markram et al., 2015; Masoli et al., 2022; Potjans and Diesmann, 2014). Some studies also incorporate more sophisticated models, e.g., including astrocytes via tripartite synapses (Lenk et al., 2020).

However, few of these approaches include energetic considerations, i.e., the dynamics of ATP hydrolysis (Kuznetsov, 2024; Wei et al., 2014). It is well-known that metabolism is involved in brain functionality: nutrients—and, in particular, oxygen (O_2_)—fuel brain specialized functions, determining the electrophysiological dynamics and brain plasticity, up to cognitive functions (Watts et al., 2018). More specifically, beyond the basic activities common to other cells (e.g., DNA and RNA synthesis), resource uptake in neurons is also dedicated to support spiking, because of the role of the Na^+^-K^+^-ATP pump in signal propagation (Attwell and Laughlin, 2001; Lennie, 2003). Since ATP dephosphorylation depends on the rate of O_2_ consumption, its dynamics can be monitored (Brosel et al., 2018; Özugur et al., 2020). O_2_ dependence is also crucial for *in vitro* slice preparations, requiring humid and well-oxygenated environment for their culturing (Sanchez-Vives et al., 2000). An analytical formulation describing O_2_-dependent firing was proposed by Wei and collaborators (Wei et al., 2014) to elucidate the mechanisms of seizure development and termination, as well as their interaction with energy metabolism. This model assumes that O_2_ variations depend on the diffusion from the bath solution and on the neuronal consumption rate for firing, but it does not consider that O_2_ can also be consumed for sustaining other metabolic functions of the cell (Attwell and Laughlin, 2001; Lennie, 2003).

The formulation proposed by Wei’s team was applied to brain tissue slices. However, O_2_ is also crucial in *in vitro* cultures: for example, in traditional monolayers, cells are inevitably exposed to different O_2_ levels when varying the amount of medium or the O_2_ boundary concentration (Al-Ani et al., 2018; Gordon and Amini, 2021; Pacitti et al., 2019; Walsh et al., 2005). Starting from Wei et al.’s model, we have developed a computational platform able to mimic the *in vitro* electrophysiological behavior of neuronal cultures at the single-cell and network level. We refer to *Digitoids* as the digitalized versions of *in vitro* neuronal monolayers obtained from dissociated neurons, in which the dependence on O_2_ concentration of network dynamics is considered. As *in vitro* networks can have different culture conditions and layouts (Antonello et al., 2022; Downes et al., 2012; Emre Kapucu et al., 2022; Hyvärinen et al., 2019), the platform is purposely designed to be modular, thus the user can generate *Digitoids* matching any type of *in vitro* neuronal network. Here we describe the theory and computational setup of *Digitoids*. For testing the performance and highlighting the crucial role of O_2_ in describing firing dynamics in neuronal cultures, we digitalized the layouts of neuron monolayers seeded on commercial micro-electrode arrays (MEAs). The O_2_-dependent model of firing and metabolism was implemented on digitalized networks to assess if a degree of similarity can be found between the *Digitoids*’ output and the corresponding experimental data from MEA recordings, comparing the predictivity of our platform to that of traditional models which neglect the dependence of firing activity on O_2_ supply. Albeit preliminary, these results highlight the significant role of O_2_ dynamics in network behaviors and thus the necessity of including energetic considerations while mathematically describing electrophysiological activity in cell cultures.

## 2 Materials and methods

### 2.1 Theory and outline of the computational platform

**FIGURE 1:**
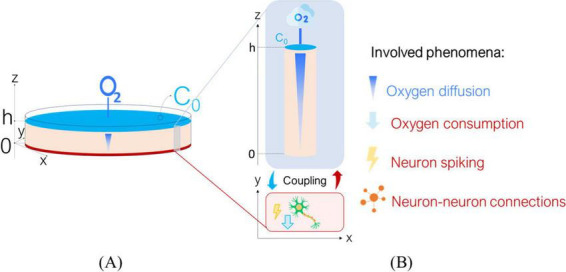
Overview of the Digitoids platform. **(A)** The *in vitro* scenario to be modeled: we describe a neuronal monolayer where cells are seeded at the bottom of a well, filled with culture medium reaching height ***h***. The top layer of culture medium interfaces with air, allowing O_2_ to diffuse and reach the cells, which consume it to sustain their metabolism and for firing. **(B)** Sketch of the coupling between O_2_ diffusion through the culture medium and single-neuron activity. We assume that single-cell dynamics can be decoupled from the network ones: a neuron lies in plane x-y and is covered by a medium column of height ***h***, where O_2_ diffuses from top to the bottom along z axis. The biophysical phenomena involved are listed on the right: O_2_ diffusion, O_2_ consumption, neuron spiking and neuron-to-neuron connections.

Figure 1. A shows the *in vitro* scenario simulated by the *Digitoids*. It is composed of a well seeded with neurons, supplied with a layer of culture medium of height *h*. The cells are assumed to be homogeneously distributed on the bottom of the well (at *z* = 0). Four phenomena occur in the system: i) O_2_ diffusion through the medium, ii) O_2_ consumption by neurons to fuel both basic cellular processes and electrophysiological activity, iii) neuron firing and iv) neural network dynamics, i.e., the transfer of electrical information via synaptic-mediated connections among cells. Considering the symmetry of the system, O_2_ diffusion can be assumed to occur only along the z axis and independently of the *x* and *y* directions (McMurtrey, 2016; Patterson and Mazurek, 2010; Place et al., 2017). Each neuron at *z* = 0 consumes O_2_ as described in the subsection *2.1.2 Single-neuron model*, generating an axial concentration gradient and a consequent downward flux. Moreover, O_2_ diffusion and reaction can be simulated as “background dynamics”, given that their characteristic times are significantly longer than those of the electrophysiological phenomena occurring on the *x*,*y* plane, where the neuron monolayer lies (Table 1). Transfer information is mainly influenced by the strength and number of synaptic connections between neurons. Thus, the O_2_-dependent single-neuron dynamics can be decoupled from those of the network as a whole. As such, the network (Figure 1A) can be considered as the integration of modules describing the O_2_ consumption—depending on its downward diffusion—as well as the firing for a single neuron (Figure 1B), modulated through the extent of its in-plane connectivity. Under these assumptions, the single-neuron metabolic and electrophysiological activity can be determined at each time step according to the O_2_ concentration perceived by the cells at *z* = 0, which is in turn updated depending on the single-neuron consumption and allows computing the diffusive flux magnitude along the medium column. On this basis, the O_2_ concentration profile at the subsequent time step can be estimated and the process iterated over time.

**TABLE 1:** Characteristic times of the phenomena involved in the single-neuron model: O_2_ diffusion—in the x direction and in the x-y plane, O_2_ consumption, neuron firing and synapses.

Phenomenon	Characteristic time (s)	References
O_2_ diffusion in z direction	∼ 0.6	(Magliaro et al., 2019)
O_2_ diffusion on x-y plane	∼ 6 10^4^	(Magliaro et al., 2019)
O_2_ consumption	∼ 31	(Magliaro et al., 2019)
Neuron firing	∼ 10^–3^	(Bean, 2007)
Synapses	∼ 2 10^–4^	(Ashwin et al., 2016; Wang et al., 2010)

From the evaluation of such characteristic times, it was possible to assume that single-neuron dynamics are decoupled from the network ones.

The computational platform was developed in Matlab (version R2023b the Mathworks Inc., Boston Massachusetts), exploiting the Simulink toolbox.

#### 2.1.1 Diffusion model

O_2_ diffusion through the culture medium is modeled as a one-dimensional phenomenon governed by the Fick’s second law:


(1) $$\frac{\partial c}{\partial t}=D\,\frac{\partial^{2}c}{\partial z^{2}}$$

where *c* (mol m^–3^) is the O_2_ concentration and *D* (m^2^ s^–1^) is the diffusion constant of O_2_ in the culture medium. Eq. (1) is solved using the finite difference method according to the initial and boundary conditions. Specifically, assuming that the well is initially filled with O_2_-saturated culture medium, the initial condition is *c*(*z*, 0) = *c*_0_, and the air-medium interface maintains a uniform and time-invariant O_2_ concentration, i.e., *c*(*h*,*t*) = *c*_0_. Note that, as neurons consume O_2_ by means of a surface reaction (i.e., they sink O_2_ as an outward flux through the well bottom), there is no volumetric reaction term to include in Eq. (1).

#### 2.1.2 Single-neuron model

The single-neuron model describes the O_2_ consumption for maintaining both vital and electrophysiological functions and the O_2_-dependent dynamics in each cell of the network as a function of the current O_2_ availability [i.e., *c*(0,*t*)] as input.

Regarding the O_2_ consumption, we assume that 75% of the available O_2_ is devoted to fuel neuronal spiking activity (namely, *c*_*f*_ = 0.75⋅*c*), and the remaining 25% (namely, *c*_*nf*_ = 0.25⋅*c*) to sustain basic cell processes (Attwell and Laughlin, 2001; Lennie, 2003). The O_2_ consumption rate of the whole neuron network (*R*(*c*), in mol m^–3^ s^–1^) can be thus expressed as:


(2) $$R\,\left(c\right)=R_{n\,f}\,\left(c_{n\,f}\right)+R_{f}\,\left(c_{f}\right)$$

where *R*_*nf*_(*c*_*nf*_) is the rate at which O_2_ is consumed for cellular and sub-cellular processes not directly linked to electrophysiological activity, and *R*_*f*_(*c*_*f*_) is the O_2_ consumption contributing to neuron firing. Specifically, *R*_*nf*_(*c*_*nf*_) can be formulated according to the Michaelis-Menten kinetics (Berger et al., 2018; Magliaro et al., 2019):


(3) $$R_{n\,f}\,\left(c_{n\,f}\right)=\frac{s\,O\,C\,R\cdot \rho_{c\,e\,l\,l\,s}\cdot c_{n\,f}}{k_{m}+c_{n\,f}}$$

where *sOCR* (mol s^–1^) is the maximal consumption rate of a single cell in the network, ρ_*cells*_ (m^–3^) is the volumetric cell density of the monolayer and *k_m_* (mol m^–3^) is the Michaelis-Menten constant, i.e., the O_2_ concentration corresponding to half saturation of the consumption rate. On the other hand, *R*_*f*_(*c*_*f*_) was described by Wei and co-workers (Wei et al., 2014) as:


(4) $$R_{f}\,\left(c_{f}\right)=\alpha \cdot I_{p\,u\,m\,p}\,\left(c_{f}\right)$$

where *I*_*pump*_ (mol m^–3^ s^–1^) is the transport rate of ions across the membrane and α (a.u.) is a conversion factor from pump transport rate to time variation of O_2_ concentration. *I*_*pump*_ is related to intracellular (subscript *i*) sodium and the extracellular (subscript *o*) potassium concentrations as in the following equation:


(5) $$I_{p\,u\,m\,p}=\frac{\rho }{1.0+\exp\,\left(\frac{25-{\left[N\,a\right]}_{i}}{3}\right)}\times \frac{1}{1.0+\exp\,\left(5.5-{\left[K^{+}\right]}_{o}\right)}$$

in which we assume that the rate ρ (mol m^–3^ s^–1^) at which the pumps transport ions across the membrane depends on the O_2_ concentration according to a sigmoidal function.


(6) $$\rho \,\left(c_{f}\right)=\frac{\rho_{m\,a\,x}}{1+{\text{exp}}^{\left(20-\frac{c_{f}}{3}\right)}}$$

In Eq. (6), ρ_*max*_ (mol m^–3^ s^–1^) is the maximal rate at which the pump operates, i.e., when the medium is fully oxygenated. Therefore, *I*_*pump*_ regulates the trans-membrane electrochemical gradient depending on the O_2_ availability, which thus influences the membrane potential *V* (mV) and the firing activity of the neuron. The Hodgkin-Huxley (HH) model is used to describe the dynamics of *V* (Hodgkin et al., 1952; Hodgkin and Huxley, 1952a):


(7) $$\frac{d\,V}{d\,t}=\frac{1}{C}\,\left(I_{e\,x\,t}-I_{N\,a}-I_{K}-I_{C\,l}\right)$$

where *C* (μF cm^–2^) is the membrane capacitance, *I*_*ext*_ (μA cm^–2^) is the external applied or synaptic current from other neurons, *I*_*Na*_,*I*_*K*_,*I*_*Cl*_ (μA cm^–2^) are the sodium, potassium and chloride currents. The latter corresponds to a leakage current, as it is mainly represented by flux of Cl^–^ ions (Hodgkin and Huxley, 1952a).

It is worth highlighting that—as in the traditional formulation of the HH model—the membrane potential *V* (Eq. 7) depends on the potassium and sodium currents *I*_*Na*_ and *I_K_*:


(8) $$I_{N\,a}\approx G_{N\,a}\,m^{3}\,p\,\left(V-E_{N\,a}\right)$$

(9) I≈GKK⁢n4⁢(V-EK)

where *m*,*p*, and *n* are activation and inactivation variables (their description is given by Supplementary Eqs. 1–7) of voltage-gated ionic channels, whose values range from *0* to *1* and define the fraction of open and closed channels throughout the membrane. For the sake of simplicity, the non-voltage-sensitive leaks were not reported. As detailed in Eqs. (8) and (9), *I*_*Na*_ and *I*_*K*_ are in turn functions of the reversal potential *E*_*Na*_ and *E_K_*, respectively, given by the Nernst equation:


(10) $$E_{N\,a}=26.64\,\ln\,\left(\frac{{\left[N\,a^{+}\right]}_{o}}{{\left[N\,a^{+}\right]}_{i}}\right)$$

(11) $$E_{K}=26.64\,\ln\,\left(\frac{{\left[K^{+}\right]}_{o}}{{\left[K^{+}\right]}_{i}}\right)$$

However, while in the HH model the intracellular concentration of sodium and the extracellular concentration of potassium are considered as constants, in this formulation they are modulated by *I*_*pump*_, which is a function of the local O_2_ concentration, as described through Eqs. (5) and (6). Thus, Nernst potentials of sodium and potassium (Eqs. 10 and 11) vary with O_2_. All the dynamics describing neuronal functioning are here assumed to occur at 37°C, corresponding to the physiological temperature for eukaryotic cells. Intracellular sodium and extracellular potassium concentrations are in turn modulated by *I*_*Na*_, *I_K_* and *I*_*pump*_, as described by the following equations (Eqs. 12 and 13):


(12) $$\frac{d\,{\left[K^{+}\right]}_{o}}{d\,t}=\gamma \,\beta \,I_{K}-2.0\,\beta \,I_{p\,u\,m\,p}$$

(13) $$\frac{d\,{\left[N\,a^{+}\right]}_{i}}{d\,t}=-\gamma \,I_{N\,a}-3.0\,I_{p\,u\,m\,p}$$

More details on the model are provided in the Supplementary Text 1 (Eqs. 8–10).

#### 2.1.3 Connectivity model

The neuronal network is generated connecting the single neurons. In this study, we implemented the neuron-to-neuron coupling via chemical synapses (Roth and van Rossum, 2009). Thus, the membrane potential of the *i*-th neuron is described by the following equation.


(14) $$\frac{d\,V_{i}}{d\,t}=\frac{1}{C^{i}}\,\left(I_{e\,x\,t}^{i}-I_{N\,a}^{i}-I_{K}^{i}-I_{C\,l}^{i}+I_{s\,y\,n}^{i}\right)$$

$I_{syn}^{i}$ in Eq. (14) is the synaptic current input to the post-synaptic neuron *i* and it is modeled as:


(15) $$I_{s\,y\,n}^{i}=\sum_{\begin{array}{c} j=1\\ j\neq i \end{array}}^{N}g_{s\,y\,n}^{j\,i}\cdot a_{i\,j}\cdot \left(E_{s\,y\,n}^{j}-V_{i}\right)$$

in which we assume that the *i*-th neuron receives inputs from *N* pre-synaptic neurons. *a*_*ij*_ is the coefficient describing the connection between vertices *i* and *j* of the adjacency matrix *A*, obtained through the Watts-Strogaz method (more details in the next Section and in Supplementary Table 2). $E_{syn}^{i}$ (mV) is the reversal potential of the synapse for the *j*-th pre-synaptic neuron and can assume the following values according to the nature of the synaptic connection (Borges et al., 2023; Wei et al., 2014).


(16) $$E_{s\,y\,n}^{j}=\left\{ \begin{array}{c} 0\,m\,V,e\,x\,c\,i\,t\,a\,t\,o\,r\,y\,c\,o\,n\,n\,e\,c\,t\,i\,o\,n\\ -80\,m\,V,i\,n\,h\,i\,b\,i\,t\,o\,r\,y\,c\,o\,n\,n\,e\,c\,t\,i\,o\,n \end{array}\right.$$

The value of the synaptic conductance $g_{syn}^{i}$ (μS cm^–2^) is modified every time the pre-synaptic neuron fires, i.e., every time *V_i_* exceeds the threshold value of 0*mV* with a positive derivative. At each spike, there is a release of neurotransmitter into the synaptic cleft, thus the synaptic conductance over time is modeled as an exponential decay:


(17) $$g_{s\,y\,n}^{j\,i}\,\left(t\right)={\bar{g}}_{syn,}^{j\,i}\cdot e^{-\frac{\left(t-t_{0}\right)}{\tau_{s\,y\,n}}}$$

where *t_0_* is the time at which the spike is fired by the pre-synaptic neuron, ${\bar{g}}_{s\,y\,n}^{j\,i}$ is the maximal conductance value and τ_*syn*_ is the decay time constant, which assumes the following values (Wei et al., 2014).


(18) $$\tau_{s\,y\,n}=\left\{ \begin{array}{c} 4\,m\,s,\mathrm{excitatory}\,\mathrm{connection}\\ 8\,m\,s,\mathrm{inhibitory}\,\mathrm{connection} \end{array}\right.$$

The synaptic dynamics are implemented in the model by updating the value of the synaptic conductance $g_{syn}^{i}$ as follows (Borges et al., 2023; Roth and van Rossum, 2009):


(19) $$g_{s\,y\,n}^{j\,i}\to g_{s\,y\,n}^{j\,i}+{\bar{g}}_{s\,y\,n}$$

(20) $$\frac{d\,g_{s\,y\,n}^{j\,i}}{d\,t}=-\frac{g_{s\,y\,n}^{j\,i}}{\tau_{s\,y\,n}}$$

where ${\bar{g}}_{s\,y\,n}=0.5$μS cm^–2^ is the intensity of the synaptic update, the same for both excitatory and inhibitory synapses. In Digitoids, 80% of neurons are excitatory and 20% inhibitory.

#### 2.1.4 Network model

It has been observed that the structure of neuronal networks in both brain tissues and cellular monolayers can be described by Small-World (SW) graphs (Antonello et al., 2022; Bettencourt et al., 2007; de Santos-Sierra et al., 2014). Specifically, a SW graph shows intermediate characteristics between a random and a regular graph, with dense clustering of neighboring vertices and short distances between pair of vertices. Indeed, *in vivo* chemical synapses typically facilitate the formation of dense local connections between neurons, thus giving rise to clusters, as well as of long-range connections allowing clusters of neurons to communicate (Bassett and Bullmore, 2006). Given a network composed of *n* vertices and *m* edges, it can be described by the metrics reported in Supplementary Table 2 (Humphries and Gurney, 2008; Watts and Strogatz, 1998).

We generated SW neural networks in a purposely developed Simulink library, which describes the wiring information through an adjacency matrix *A*, usually used to represent inter-neuron connections (de Santos-Sierra et al., 2014; Poli et al., 2015; Shefi et al., 2002). Starting from the number of vertices and edges and the metrics characterizing the networks, *A* can be obtained using the Watts-Strogatz method (Chen et al., 2007). Each coefficient of the matrix describes the connectivity between vertex *i* and *j*. Specifically, *a*_*ij*_ = 1 if an edge exists from vertex *i* to vertex *j*, otherwise it is *0*. We thus exploited such adjacency matrices to create connections between neurons, defined by chemical synapses (Eqs. 14–20). Both the O_2_ diffusion and the single-neuron models were integrated in the library, which allows defining: (i) the initial and boundary O_2_ concentration c_0_ at the air-medium interface, (ii) the height of the medium *h*, and (iii) the metabolic and firing parameters of the neuron.

### 2.2 Impact of oxygen on single-neuron activity

For assessing the influence of O_2_ availability on firing, the single-neuron model coupled with O_2_ diffusion was first computed using stepwise variations of both (i) the boundary concentration of O_2_ from 0.2 mol m^–3^ (i.e., the maximum available oxygen concentration in water) to 0.04 mol m^–3^ [i.e., the critical oxygen concentration for cell survival (Berger et al., 2018)] and (ii) the culture medium height *h* from 0.1 to 3 mm, based on the conditions usually used for neuron electrophysiological recordings (Ballesteros-Esteban et al., 2023; Negri et al., 2020; Scelfo et al., 2012). All the parameter combinations were simulated for 20 s (variable step solver “ode15s” by Simulink) and are summarized in Table 2.

**TABLE 2:** Values of the parameters simulated in the single-neuron configuration.

h (mm)	c_0_ (mol m^–3^)
3	0.2
2	0.16
1	0.12
0.5	0.08
0.1	0.04

Every combination of the two parameters – medium height ***h*** and boundary O_2_ concentration ***c*_0_**−was tested, for a total of 25 configurations in the single-neuron model, to assess and characterize the influence of these parameters in shaping the resulting electrophysiological activity.

### 2.3 Analysis of single-neuron membrane potential

**FIGURE 2:**
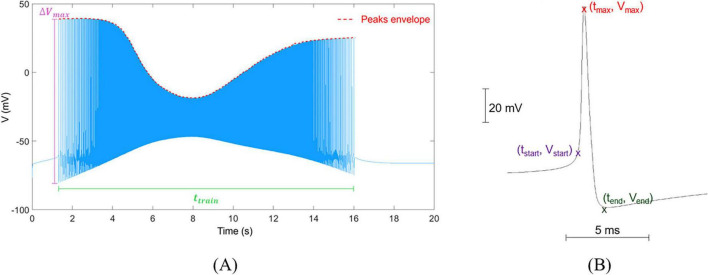
Visual representation of trains and single spikes, and their metrics. **(A)** Example of a spike train simulated for a single neuron, in which membrane voltage V (mV) varies in time (s). The plot reports the parameters used to calculate AR and DR: **Δ** ***V_max_*** is the maximal peak-to-peak amplitude, ***t_train_*** is the time duration of the train, and the peak envelope is used to calculate the average value of its first derivative, α. **(B)** Waveform of a single spike isolated from the train with indication of reference points for calculating its electrophysiological characteristics (***v_pp_***, ***rr*** and ***fr***).

To characterize how the shape of the spike trains and the single-spike waveforms are influenced by the different combinations of *c_0_* and *h*–and, thus, by the overall O_2_ availability within the system—we defined two new metrics: the Aspect Ratio (*AR*, expressed in logarithmically-scaled mV s^–1^) and the Dissipation Rate (*DR*, expressed in s^–1^), defined as follows:


(21) $$A\,R=\log_{10}\,\frac{\Delta \,V_{m\,a\,x}}{t_{t\,r\,a\,i\,n}}$$

(22) $$D\,R=\frac{\alpha }{\Delta \,V_{m\,a\,x}}$$

where Δ*V*_*max*_ is the peak-to-peak amplitude of the highest spike in the train, *t*_*train*_ is the time duration of the train and α (in mV s^–1^) is the average value of the first derivative of the envelope of the peaks in the train. *t*_*train*_ was expressed as the difference between the end and start times *t*_*end*_ and *t*_*start*_, identified as the time at which the first derivative of the signal is equal to 0 and the time at which the signal amplitude > −60 mV (Di Florio et al., 2022; Wilson and Emerson, 2002), respectively. Figure 2A reports a typical spike train, and a graphical representation of the quantities used to calculate *AR* and *DR*.

We separately assessed the correlation of each of the three metrics–*t*_*train*_, AR and DR—with the boundary O_2_ concentration *c_0_* and the medium height *h* by computing the non-parametric Spearman coefficient (significance level of 0.05).

The shape of single spikes was also evaluated, calculating the peak-to-peak amplitude (*v*_*pp*_ = *v*_*max*_−*v*_*min*_, expressed in mV), rise rate (*rr* = (*v*_*max*_−*v*_*start*_)/(*t*_*max*_−*t*_*start*_), in mV s^–1^) and fall rate (*fr* = (*v*_*max*_−*v*_*end*_)/(*t*_*max*_−*t*_*end*_) in mV s^–1^), where *t*_*max*_ is calculated as the time corresponding to the maximum of the spike (Figure 2B; Ghaderi et al., 2018; Zaitsev et al., 2012).

Finally, to describe the features of the spikes fired by single neurons as a function of the balance between diffusive O_2_ supply and its consumption by the neurons irrespective of the specific setup of the simulation, we exploited the Thiele Modulus, Φ^2^. Specifically, Φ^2^ is defined as the ratio between the characteristic diffusion (τ_*d*_) and reaction (τ_*r*_) times. Since metabolism and firing occur simultaneously in the neuron domain, the reaction dynamics is driven by the faster of the two phenomena. Given that the reaction is described by the sum of two rates (Eq. 2), Φ^2^ can be formulated as follows:


(23) $$\Phi^{2}=\frac{\tau_{d}}{\tau_{r}}=\tau_{d}\,\left(\frac{1}{\tau_{n\,f}}+\frac{1}{\tau_{f}}\right)=\frac{\tau_{d}\,\left(\tau_{n\,f}+\tau_{f}\right)}{\tau_{n\,f}\cdot \tau_{f}}$$

where τ_*nf*_ and τ_*f*_ indicate the characteristic times of basal and firing-related O_2_ consumption, respectively. Refer to Supplementary Text 2 for further details on the derivation of Eq. (23). The shape metrics of the spike trains–*AR* and *DR*−were then also evaluated as a function of Φ^2^.

### 2.4 Assessment of digitoids performance

#### 2.4.1 Digitoids versus experimental data

**FIGURE 3:**
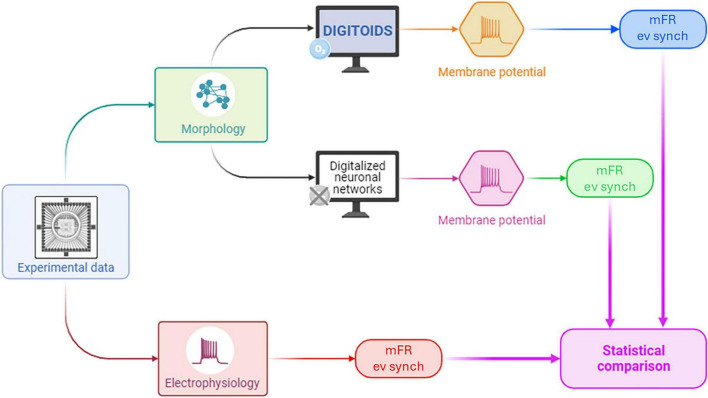
The pipeline adopted for assessing the performance of Digitoids using experimental data. Experimental topological parameters from neurons cultured on MEAs were used to build the *Digitoids* and the HH (i.e., O_2_-independent models) neuronal networks. The *Digitoids* and HH models were simulated, and their membrane potentials were processed in the same way as the experimental electrophysiological data to extract mFR. Finally, mFRs from the two computational models – *Digitoids* and HH – and experimental neuronal cultures were statistically compared to assess whether a similarity exists.

*Digitoids* performance was evaluated using the experimental data presented in Ballesteros-Esteban et al. (2023), following the pipeline shown in Figure 3. In ref. (Ballesteros-Esteban et al., 2023), the authors describe the morphology and the electrophysiological activity of neuron networks *in vitro*. The network activity was recorded via a MEA, and the mean Firing Rate (mFR) as well as the event synchronization were extracted. The topological evolution of the networks was mapped to a network graph, where neurons are represented as vertices and their physical connections as edges, and the SW metrics were defined. Their experimental setup and SW metrics are detailed in Supplementary Tables 1, 2, and a more in-depth description of the experimental set-up and procedures is provided in Supplementary Text 4. In our work, measurements from *Day In Vitro* (DIV) 11 to DIV 16—i.e., when the network exhibits a SW layout (Ballesteros-Esteban et al., 2023) —were exploited, without the intention of mapping the temporal evolution of the *in vitro* neuronal cultures. Given the number of vertices and edges for those DIVs reported in Ballesteros-Esteban and co-workers and the metrics characterizing the networks, the adjacency matrix was obtained through the Watts-Strogatz method, setting the rewiring probability to 0.5 (Watts and Strogatz, 1998). Three SW graphs were obtained for each DIV considered. The outcoming connectivity models are sparse (i.e., the number of edges is less than the possible number of edges in the order of O(q), where q is the total number of vertices), with a mean edge density (defined in Supplementary Table 2) of 2.5%, in consistence with previously reported experimental values (Antonello et al., 2022; Downes et al., 2012).

The SW layouts and the adjacency matrices were used to generate the corresponding *Digitoids*. The layouts, along with their number of vertices and edges, are reported in Supplementary Table 3, while the model parameters are listed in Table 3. The same SW layouts were used to build *in silico* neuronal networks where the traditional HH model was implemented instead of the O_2_-dependent one, described in Section 2.1.2. The single-neuron description was obtained by imposing the membrane pump to work optimally, i.e., with fixed pump rate ρ_*max*_ (Table 3). The current components of the model are the same of the single-neuron model (Section 2.1.2)—i.e, *I*_*Na*_, *I_K_* and *I*_*Cl*_−−consistently with the model developed by Wei and co-workers (Wei et al., 2014). The neurons in the computational network models are spontaneously active due to potassium concentration in the bath. All the network models were simulated for 20 s with the variable-step solver “ode15s” of Simulink, with maximal step size of 0.4.

**TABLE 3:** Values of the parameters used in the model.

Model parameter	Value	References
Diffusion constant (D)	$2.69\cdot 10^{-9}\,\frac{m^{2}}{s}$	(McMurtrey, 2016)
Oxygen Consumption Rate per cell (sOCR)	$5.28\cdot 10^{-14}\,\frac{g}{s\cdot c\,e\,l\,l}$	(Huchzermeyer et al., 2013)
Cell density (ρ_*cells*_)	$1.2\cdot 10^{13}\,\frac{c\,e\,l\,l}{m^{3}}$	(Ballesteros-Esteban et al., 2023)
Michaelis-Menten constant (*k_m_*)	$9.79\,\frac{m\,g}{l}$	(Huchzermeyer et al., 2013)
Conversion factor from pump current to oxygen concentration (α)	0.17	(Wei et al., 2014)
Conversion factor current to concentration (γ)	$0.04445\,\left(\frac{m\,M}{s}\right)/\left(\frac{\mu \,A}{c\,m^{2}}\right)$	(Wei et al., 2014)
Ratio to intra/extracellular volume (β)	7	(Wei et al., 2014)
Maximal Na-K pump rate (ρ_*max*_)	$1.25\,\frac{m\,o\,l}{m^{3}\cdot s}$	(Wei et al., 2014)
Membrane capacitance (*C*)	1μ*F*/*cm*^2^	(Wei et al., 2014)
Maximal sodium conductance (*G*_*Na*_)	30*mS*/*cm*^2^	(Wei et al., 2014)
Maximal potassium conductance (*G_K_*)	25*mS*/*cm*^2^	(Wei et al., 2014)
Reversal potential of synapses, $E_{syn}^{j}$	0*mV*, if excitatory	(Borges et al., 2023; Wei et al., 2014)
	−80*mV*, if inhibitory	
Time constant of synapses, τ_*syn*_	4*ms*	(Borges et al., 2023; Wei et al., 2014)
	8*ms*	
Synaptic update, ${\bar{g}}_{s\,y\,n}$	0.5μ*Scm*^−2^	(Borges et al., 2023)

This table reports all the constants adopted in the model described in this work.

#### 2.4.2 Impact of oxygen on network-level activity

Six *Digitoids* (with SW layout size described in Supplementary Table 3) were developed and simulated to explore the effects of O_2_ deprivation on the network activity. For this purpose, the six *Digitoids* were first simulated in normal oxygenation conditions for cell culture, i.e., considering a boundary concentration *c*_0_ = 0.2*mM*. Then, the same networks were simulated lowering *c_0_* to 0.04*mM*, i.e., the threshold O_2_ concentration ensuring physiological cell functioning and survival (Berger et al., 2018).

### 2.5 Statistical analysis

Statistical analyses were performed using GraphPad Prism 8 (GraphPad Software, Boston, Massachusetts United States) to identify any significant differences between the mFR of the computational models and the experimental data. Thus, firstly, the distributions of mFR of the O_2_-dependent firing in *Digitoids*, the mFR experimentally measured in cultured neurons and the mFR values from the traditional HH model were tested for normality, by adopting the Shapiro-Wilk test (α = 0.05). Since the distributions were not Gaussian, the non-parametric Kruskal-Wallis test was used (α = 0.05). To compare mFR and event synchronization between the *Digitoids* simulated in normal (i.e., *c*_0_ = 0.2*mM*) and O_2_ deprivation (i.e., *c*_0_ = 0.04*mM*) conditions, the Mann-Whitney test was instead adopted (α = 0.05).

## 3 Results

### 3.1 Dependence of firing on oxygen availability

**FIGURE 4:**
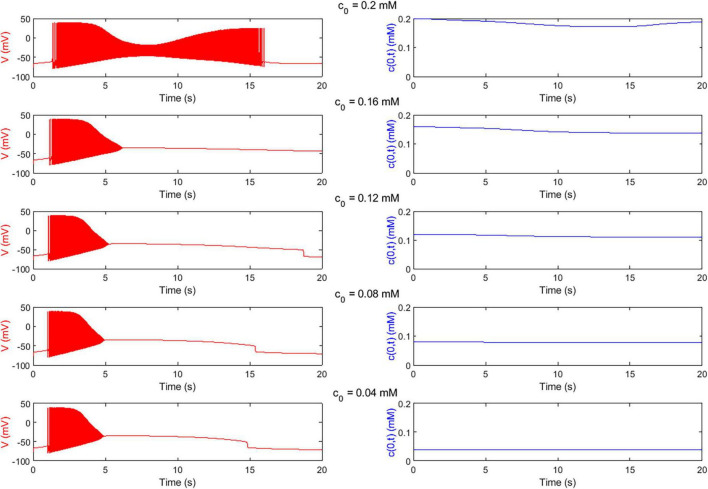
O_2_-dependent electrophysiological activity of single neurons predicted by *Digitoids*. Membrane potential (***V***, left column) and O_2_ concentration at the neuron level (***c***(**0**,***t***), right column) over the first 20 s of the simulation of the single-neuron model with ***h*** = **0.1 *mm*** for different values of boundary O_2_ concentration ***c*_0_** (reported in Table 2). In the upper panel, the output of the configuration with highest O_2_ availability is depicted. The neuron is able to fire a long train of action potentials. In correspondence of the firing, c(0,t) decreases because O_2_ is consumed by the cell to sustain metabolism and electrical activity.

Figure 4 shows examples of the outcome of the *Digitoids*, i.e., the neuron membrane potential and the O_2_ concentration at the cell level (z = 0) taken over a time window of 20 s for different values of the boundary O_2_ concentration. As expected, the plots indicate that single neurons exhibit an O_2_-dependent firing, with reduced activity when the local concentration decreases. Indeed, when the neuron fires, the Na^+^-K^+^-ATP pump is activated, thus O_2_ is consumed (Eqs. 2–4), and its concentration at *z* = 0 decreases. Longer spike trains are generated if O_2_ availability is high.

For what concerns the sensitivity of the shape metrics to the parameters *c_0_* and *h*, plots are reported in Supplementary Figures 5, 6. Specifically, Supplementary Figure 5 graphically depicts the dependence of *t*_*train*_, AR and DR (Eqs. 21 and 22) on *c_0_* for each of the tested medium heights, while Supplementary Figure 6 reports their dependence on *h* parametrized with respect to *c_0_*. From the visual analysis of the plots, a monotonic relation can be identified between the parameters *c_0_* and *h*–which set the availability of O_2_ over time to the neuron—and the train metrics. This suggests that the neuron is able to fire longer trains of action potentials when the O_2_ availability in the system is not a limiting factor, i.e., with highest *c_0_* and lowest *h*. Furthermore, the Spearman correlation coefficient *r* was computed to provide a quantitative means of such dependencies. Numerical values of *r* are reported in Supplementary Tables 4–9 together with corresponding *p*-values. All the metrics display significant correlation with *c_0_*, while they significantly correlate with the medium height only when boundary O_2_ is maximal. Indeed, the single-neuron output is more sensitive to growing medium heights when O_2_ availability is not limited yet by reduced air saturation, that is *c_0_* < 0.2 mM. Otherwise, supply constraints due to the increased diffusive path do not significantly affect the duration of spike trains.

**FIGURE 5:**
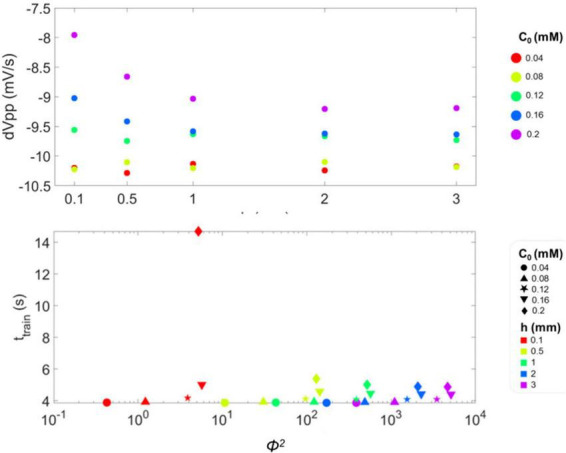
**(A)** Slope of the peak-to-peak amplitude of single spikes (***dV_pp_***) calculated for the last four points of ***v_pp_*** in the spike train and plotted as a function of medium height ***h***. For the three lowest values of ***h* − 0.5, 1 *and* 2 *mm* −**, the value of ***dV_pp_*** is more negative when ***c*_0_** is lower. This means that the train of action potentials fired by the single-neuron model is terminated with a steeper slope, i.e., faster with respect to conditions of higher ***c*_0_**. **(B)** Duration of the spike train, ***t_train_***, as a function of **Φ^2^**, whose values are reported in log-scale. Datapoints correspond to the considered combinations of ***c*_0_** and ***h***, reported in Table 2: different symbols correspond to different values of ***c*_0_**, while colors to ***h***.

Moreover, single spikes were identified for each combination of *h* and *c_0_*; for each spike, *v*_*pp*_, *rr* and *fr* were calculated, and their trend over time are shown in Supplementary Figures 1–3. At the beginning of the simulation (i.e., when O_2_ availability is high), *v*_*pp*_ values appear independent of *h* and *c_0_* (see Supplementary Figure 1). Then, *v*_*pp*_ decreases over time with a rate depending on *c_0_*. In particular, we observed that the rate at which *v*_*pp*_ decreases at the end of the spike train is higher for the lower values of *c_0_*. This is reported in Figure 5A, where the slope of *v*_*pp*_ (*dV*_*pp*_) over the last four time points considered in the simulation is shown to better highlight the dependence on the different values of *h* and *c*_0_.

Figure 5B depicts *t*_*train*_ as a function of Φ^2^. Notably, *t*_*train*_ is sensitive to the level of O_2_ available to the neuron, as reported in Bean (2007), since it decreases with higher Φ^2^ (that is with lower *c_0_* and higher *h*). This implies that firing is a diffusion-limited phenomenon, which is suppressed when it cannot be energetically sustained due to O_2_ depletion (Nieber, 1999; Pires Monteiro et al., 2021; Santiago et al., 2023). Moreover, the dispersion of *t*_*train*_ values becomes narrower with increasing Φ^2^, indicating that the firing threshold is governed by O_2_ availability, which is in turn increasingly limited by diffusion as *h* increases.

The same trends with respect to Φ^2^ are observable for *DR* and *AR*−see Supplementary Text 3 and Supplementary Figure 4 for details.

### 3.2 Performance of the digitoids

**FIGURE 6:**
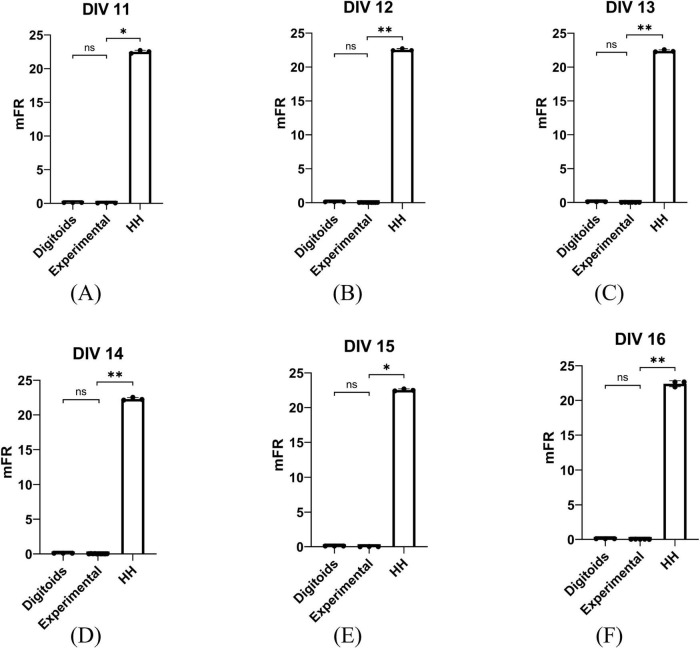
Statistical comparison between mFR (spikes s^–1^) values obtained from experimental measurements, the Digitoids and the HH model for the six different Day *In Vitro* (DIV) considered for analyses: **(A)** DIV 11, **(B)** DIV 12, **(C)** DIV 13, **(D)** DIV 14, **(E)** DIV 15, **(F)** DIV 16. At each DIV, mFR calculated from *Digitoids* and Experimental data exhibits no tatistically significant differences, while Experimental and *Digitoids* are always different. *p < 0.05, **p < 0.005.

Figure 6 shows the mFR obtained from: (i) the experimental *in vitro* recordings reported in Ballesteros-Esteban et al. (2023); (ii) the output from the corresponding *Digitoids*; (iii) the firing activity of the network with the same topological layout but the traditional formulation of electrophysiology according to the HH model. For all the DIV considered, no statistically significant differences were found between the mFR of *Digitoids* and the corresponding experimental data. On the other hand, the values of mFR of the traditional O_2_-independent HH model were significantly different if compared to both the *in vitro* observations and *Digitoids* predictions. The associated p-values are reported in Supplementary Table 10.

**FIGURE 7:**
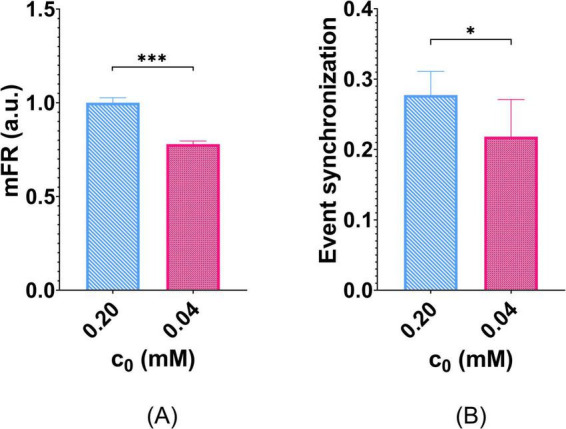
Effects of O_2_ deprivation on network activity. Six *Digitoids* were simulated with normal O_2_ and with deprived O_2_ in culture medium to compare their mFR. **(A)** mFR is significantly reduced when the networks are simulated in O_2_ deprivation conditions. mFR values are reported in a.u. since they were normalized for the average value calculated in normoxic conditions (i.e., *c*_0_ = 0.20*mM*). ***p < 0.001. **(B)** O_2_ deprivation also reduces the event synchronization of the networks, with statistically significant differences with respect to the normoxic condition. *p < 0.05.

Further, the whole-network effect of O_2_ deprivation on mFR predicted by *Digitoids* is shown in Figure 7A. When accounting for reduced O_2_ availability (*c*_0_ = 0.04*mM*), *Digitoids* coherently predicted significantly lower mFR than that obtained for *c*_0_ = 0.2*mM*. The event synchronization was also evaluated in such conditions. Also in this case, O_2_ deprivation lowers the predicted synchronization values, with significant differences with respect to values predicted by the *Digitoids* with normoxic conditions (Figure 7B).

Supplementary Figure 7 depicts an example of the event synchronization calculated from one of the simulated *Digitoids*.

## 4 Discussion

O_2_ levels are crucial to neuronal function *in vitro:* they significantly affect viability, oxidative stress and mitochondrial function (Zhu et al., 2012). However, the influence of O_2_ on *in vitro* electrophysiological behavior is often neglected. In this work, we developed a computational platform—*Digitoids*—able to replicate a neuronal network *in vitro*. *Digitoids* embeds a model of neuron firing in which the O_2_ dynamics of diffusion and consumption are introduced and coupled with ionic transport across the cell membrane. The novelty of the proposed model resides in the coupling of O_2_ diffusion and consumption dynamics with neuronal electrical activity. Thanks to this approach, different culture conditions and layouts can be replicated obtaining descriptions of O_2_-dependent activity tailored on the specific system under study.

To demonstrate the importance of O_2_ in neuron firing, we computed different metrics of the spike train as well as of single spikes and assessed their dependency on O_2_ availability. Overall, the observed trends confirm that the electrophysiological behavior of single neurons is modulated by O_2_ supply. These results are supported by the significant correlation between the train metrics and the boundary O_2_ concentration, *c_0_*. Interestingly, neuron firing was found to be less sensitive to O_2_ fluctuations in conditions of limited resource availability (i.e., for high Φ *^2^* values). Indeed, reduced—or even non-significant—correlation coefficients of spike train characteristics with medium height are found when boundary O_2_ does not correspond to air saturation (i.e., *c* = 0.2 mM).

This behavior can be explained considering that reduced O_2_ hinders the homeostatic maintenance of ion concentrations between the intra and extracellular environments, which is responsible for sustaining the electrical activity of the neuron, as reported for both brain slices and *in vitro* cultures exposed to hypoxia (Brisson et al., 2013; Fiskum et al., 2021; Pires Monteiro et al., 2021; Spong et al., 2016; Zanelli et al., 2015). Under these conditions, the Na^+^-K^+^-ATP pump lacks sufficient resources to fuel ion transport, and thus firing decreases or even ceases (Nieber, 1999). The preliminary results obtained simulating O_2_ deprivation at the network level corroborate this evidence, suggesting that cells reduce their electrical activity and synchronization than in standard oxygenation at both the single-neuron and whole-network scale. These results are consistent with studies which reported reduced firing rate of cultured neurons when exposed to hypoxia (Fiskum et al., 2021; Hofmeijer et al., 2014).

As a first preliminary assessment of the goodness of *Digitoids* predictions, we compared the simulated firing rate to that measured in neuronal networks seeded on commercial MEAs. No statistically significant differences were found between the experimentally measured mFRs and those predicted by *Digitoids*. Additionally, we compared the mFRs observed *in vitro* to predictions by the classic HH model applied to the same network layouts. The results are significantly different, highlighting that the mutual influence between local O_2_ concentration and the ion pump activity affects electrophysiological dynamics, as also captured by the analyses performed on the single-neuron output. It is worth highlighting that for *Digitoids* and experimental data the mFR is much lower than in the traditional HH model. In the latter, the initial values of simulation parameters (described in Section 2.4) indeed induce neurons to fire longer trains of APs, which are not limited by reduced O_2_ availability. Including O_2_ dynamics instead allows *Digitoids* to mimic its potential deprivation due to neuronal uptake, hindering the cross-membrane transport of ions as the energetic demand of membrane pumps cannot be satisfied.

The platform is designed to be modular and adaptable to different culture conditions by tuning the cell metabolic and electrophysiological parameters. More complex models of neuronal cultures, (e.g., co-cultures) can be developed by adding different single-cell blocks to the Simulink library to mimic other neural phenotypes. Following the approach described in Callegari et al. (2023), three-dimensional (3D) neuronal constructs can also be built by overlaying monolayers on each other. Thus, *Digitoids* can be extended to the simulation of neurospheres and cerebral organoids (Poli et al., 2019), supporting the investigation of their biophysical mechanisms. These constructs are particularly susceptible to O_2_ availability, as its depletion can lead to the formation of non-viable cores, hindering the development of mature traits and of a 3D neural network (Poli et al., 2019).

It is important to note that the experimental data used for comparison were derived from insect neurons, while the model parameters are typical of mammalian neurons. Nevertheless, the fundamental mechanisms underlying spike generation are similar across different species (Spong et al., 2016), and invertebrates are widely used to advance our understanding of more complex organisms (Newcomb et al., 2023; Sattelle and Buckingham, 2006). A more in-depth validation of our platform would require parallel recordings of electrophysiological and O_2_ dynamics in *in vitro* neurons. Specifically, perturbations will be added in the model and the predicted output will be compared to an experimental setting where the same perturbation is introduced (e.g., incubator O_2_ level drop). Furthermore, future effort will be carried out to integrate in the model also the O_2_ demand of synaptic activity (Faria-Pereira and Morais, 2022). For what concerns the parameters specific of the electrophysiological model, they will be tuned to better fit the recorded electrophysiology of *in vitro* neurons.

To proof the feasibility of using *Digitoids*, we exploited topological and electrophysiological data acquired on low density cultured networks. Thus, the simulated networks involve a relatively limited number of neurons and connections. To further expand the relevance of this work, larger networks can be developed and simulated. Larger-sized *Digitoids* can be developed with the same approach described in this work (Section 2.4.1) by defining a bioinspired (i.e., based on biologically observed features) adjacency matrix to layout the spatial distribution of single neurons and their connections within the Simulink framework.

In conclusion, this work represents a promising first step towards creating “digital twins” of *in vitro* neuronal networks. The approach implemented in *Digitoids* can be exploited for gaining important insights into brain pathophysiology. As an example, stroke and ischemia are characterized by low O_2_ levels, which lead to cognitive decline, neuronal damage and cell death (Klein Gunnewiek et al., 2020; Radenkovic et al., 2024; Voogd et al., 2023). In addition, neurodegeneration is known to be intimately linked to mitochondrial—and thus bioenergetic—dysfunctions (Bustamante-Barrientos et al., 2023). Hence, *Digitoids* hold the potential to support, or even replace, primary neuronal cultures, as they are cost-effective, have a longer lifespan and allow high-throughput experiments that would be unfeasible *in vitro* (Velasco et al., 2020). Ongoing efforts include further model validation through detailed O_2_ and electrophysiological measurements, as well as expanding the model to include additional modules for different neuron types and 3D networks.

## Data Availability

The original contributions presented in the study are included in the article/Supplementary material, further inquiries can be directed to the corresponding authors.
