# Supplementary material for: Digitoids: a novel computational platform for mimicking oxygen-dependent firing of neurons in vitro
Source: Front Neuroinform. 2025 Jul 1;19:1549916. doi: 10.3389/fninf.2025.1549916 (PMC12259620; doi:10.3389/fninf.2025.1549916)
Supplement: Supplementary file 1 [file Data_Sheet_1.docx]

Supplementary Material

# Supplementary Information text

## Single-neuron model

The dynamics of the activation and inactivation variables – $m$, $p$, and $n$ – of the ionic channels are expressed in the form

|  | $\frac{dx}{dt}=\alpha_{x}\left( 1-x \right)-\beta_{x}$ | (1) |
| --- | --- | --- |

with $x\in\left\{ m, p, n \right\}$. The rate constants $\alpha$ and $\beta$ (s^-1^) related to $m, h, n$ are functions of $V$ (mV) and are defined as follows (Wei et al., 2014):

|  | $\alpha_{m}=\frac{0.32(V+54)}{1-\exp\left[ -{(V+54)}/4 \right]}$ | (2) |
| --- | --- | --- |
|  | $\beta_{m}=\frac{0.28(V+27)}{\exp\left[ {(V+27)}/5 \right]-1}$ | (3) |
|  | $\alpha_{p}=0.128\exp\left[ -{(V+50)}/{18} \right]$ | (4) |
|  | $\beta_{p}=\frac{4}{1+\exp\left[ -{(V+27)}/5 \right]}$ | (5) |
|  | $\alpha_{n}=\frac{0.032(V+52)}{1-\exp\left[ -{(V+52)}/5 \right]}$ | (6) |
|  | $\beta_{n}=0.5\exp\left[ -{(V+57)}/{40} \right]$ | (7) |

The expressions of the sodium, potassium and chloride currents (μA cm^-2^) are the following:

|  | $I_{Na}=G_{Na}m^{3}p\left( V-E_{Na} \right)+G_{NaL}(V-E_{Na})$ | (8) |
| --- | --- | --- |
|  | $I_{K}=G_{K}n^{4}\left( V-E_{k} \right)+G_{KL}\left( V-E_{K} \right)$ | (9) |
|  | $I_{Cl}=G_{ClL}(V_{E_{Cl}}-E_{Cl})$ | (10) |

where $G$ (mS cm^-2^) represents the conductance of the voltage-gated channel and the subscript $L$ indicates the non-voltage-gated leak contributions to the currents. The values of $G_{Na}$ and $G_{K}$ are given in Table 3 of the Main text. The leak conductances have the following values: $G_{NaL}=0.0175 mS/cm^{2}$, $G_{KL}=0.05 mS/cm^{2}$ and $G_{ClL}=0.05 mS/cm^{2}.$ $E_{Cl}=26.64\ln\left( \frac{\left[ Cl^{-} \right]_{i}}{\left[ Cl^{-} \right]_{o}} \right)$, with $\left[ Cl^{-} \right]_{i}=6 mM$ and $\left[ Cl^{-} \right]_{o}=130 mM$.

$I_{Na}$ and $I_{k}$, along with $I_{pump}$ (Eq. 5 in the main text) in turn modulate the intracellular sodium and the extracellular potassium concentrations, as described by the following equations:

|  | $\frac{d\left[ K^{+} \right]_{o}}{dt}=\gamma\beta I_{K}-2.0\beta I_{pump}$ | (11) |
| --- | --- | --- |
|  | $\frac{d\left[ Na^{+} \right]_{i}}{dt}=-\gamma I_{Na}-3.0I_{pump}$ | (12) |

## Thiele modulus

$$\Phi^{2}=\frac{\tau_{d}}{\tau_{r}}$$

Where the characteristic time of diffusion and reaction are:

$$\tau_{d}=\frac{h^{2}}{D}$$

$$\tau_{r}=\frac{1}{\frac{1}{\tau_{nf}}+\frac{1}{\tau_{f}}}$$

The complete expressions of the characteristic time of metabolism and firing dynamics are:

$\tau_{nf}=\frac{k_{m}+c_{nf}}{\rho_{cells}\cdot sOCR}$ with $c_{nf}$ defined as $c_{nf}=0.25\cdot c_{0}=\frac{1}{4}{\cdot c}_{0}$

$\tau_{f}=\frac{c_{f}}{\alpha\cdot\rho}=\frac{1+\exp\left( 20-\frac{c_{f}}{3} \right)}{\alpha\cdot\rho}$ with $c_{f}$ defined as $c_{f}=0.75\cdot c_{0}=\frac{3}{4}\cdot c_{0}$

From the above expressions, we obtain the following for $\tau_{r}$:

$$\tau_{r}=\left( \frac{\rho_{cells}\cdot sOCR}{k_{m}+c_{nf}}+\frac{\alpha\cdot\rho}{1+\exp\left( 20-\frac{c_{f}}{3} \right)} \right)^{-1}=\left( \frac{\rho_{cells}\cdot sOCR}{k_{m}+\frac{c_{0}}{4}}+\frac{\alpha\cdot\rho}{1+\exp\left( 20-\frac{\frac{3\cdot c_{0}}{4}}{3} \right)} \right)^{-1}=\left( \frac{4\cdot\rho_{cells}\cdot sOCR}{{4\cdot k}_{m}+c_{0}}+\frac{\alpha\cdot\rho}{1+\exp\left( 20-\frac{c_{0}}{4} \right)} \right)^{-1}$$

So, the complete expression of $\Phi^{2}$ is:

$$\Phi^{2}=\frac{\tau_{d}}{\tau_{r}}=\frac{h^{2}}{D}\cdot\left( \frac{4\cdot\rho_{cells}\cdot sOCR}{{4\cdot k}_{m}+c_{0}}+\frac{\alpha\cdot\rho}{1+\exp\left( 20-\frac{c_{0}}{4} \right)} \right)^{-1}$$

## Further considerations on the dependence of firing on oxygen availability

Both the rise rate ($rr$, Supplementary Figure 2) and the fall rate ($fr$, Supplementary Figure 3) of the single spike display trends over time consistent with those observed for $V_{pp}$ (Supplementary Figure 1). At the beginning of the simulation, the mean values of such indices - corresponding to different configurations of the *Digitoids* - cannot be distinguished, then configurations with the highest $c_{0}$ maintain higher values of $rr$ and lower values of $fr$ as, in this case, neurons are supplied with more O_2_. As observed in terms of $V_{pp}$, the neuron appears to be more sensitive to $c_{0}$ when the height of the culture medium is low. In addition, it is worth noting that, for both indices, the widest dispersion (i.e., highest standard deviation) corresponds to the last timepoint evaluated. Indeed, the last time interval in which spike metrics are evaluated is at the end of the spike train, thus spikes exhibit sudden changes in their form.

## Description of the *in vitro* cultured neuronal networks

In ref. (Ballesteros-Esteban et al., 2023), the authors describe the morphology and electrophysiological activity of 19 *in vitro* insect neuronal networks cultured on MEA chips with 120 electrodes at a density of 1200 cells/chip at different time points (i.e., days in vitro – DIV). The cells were cultured with 2 mL of medium, corresponding to a height of 3 mm over a plate with an area of $\sim550 mm^{2}$. The topological evolution of the networks was examined by acquiring images through an inverted phase-contrast microscope. The images were segmented to identify the so-called network graph, where neurons are represented as vertices and their physical connections as links, and the SW metrics were thus defined. The network electrical activity, recorded via a MEA (Multichannel Systems, 120MEA200/30iR-Ti-pr), is described in terms of mFR and event synchronization. For evaluating mFR, firstly, each signal recorded by the electrodes of the MEA is processed for detecting spikes, with an adaptive threshold of ±6σ (where σ is the standard deviation of the signal in the considered window) calculated over a sliding window of 1 s, then the electrode firing rate is calculated as the ratio between the total number of spikes per active electrode (i.e., more than 5 spikes were detected in the 25-min recording segment) and the recording time. Finally, the mFR of the network is defined as the average firing rate over the whole MEA. Event synchronization was assessed between each pair of electrodes as the correlation between the time series of their spikes. Firstly, spike times were divided into time bins and each spiking sequence was transformed into a binary series: the *i*-th element of the series is 1 if the considered electrode has a spike within time bin $i$, otherwise it is 0. Then, for each pair of electrodes, the correlation was assessed, obtaining a correlation matrix with dimensions corresponding to number of electrodes.

# Supplementary Figures and Tables

## Supplementary Figures


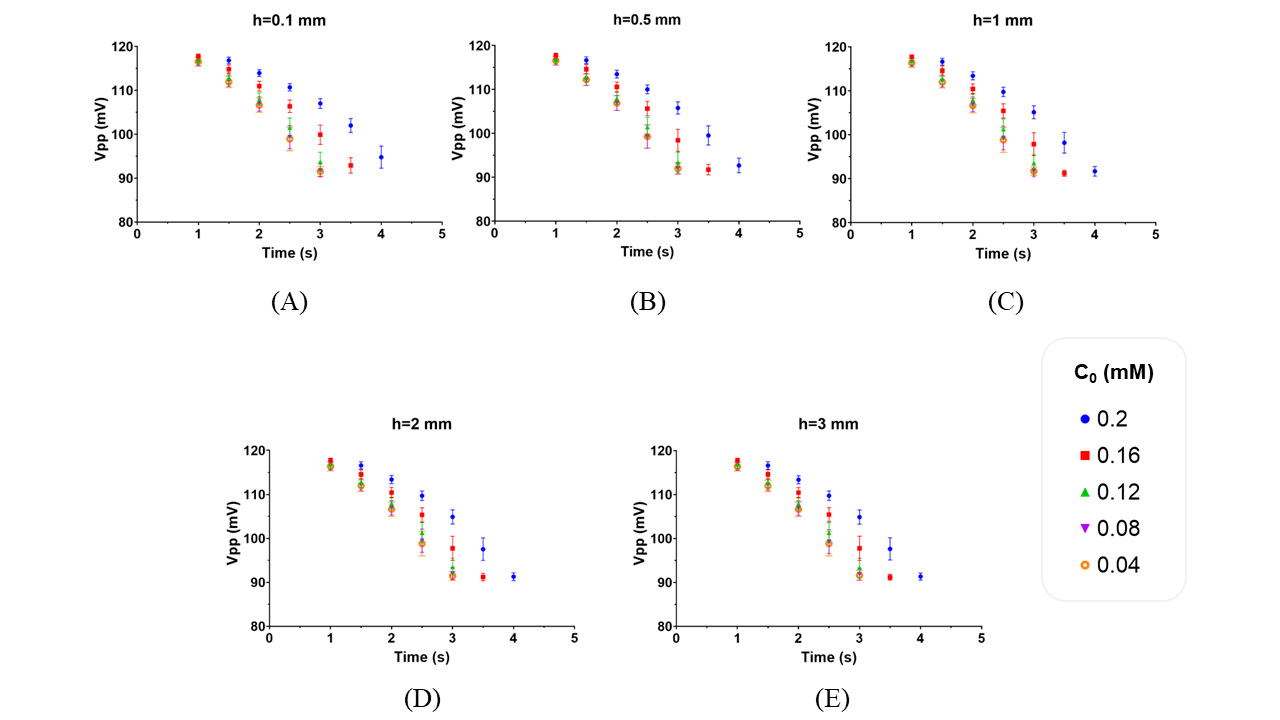


Supplementary Figure 1. Peak-to-peak amplitude over time as a function of $\boldsymbol{c}_{\boldsymbol{0}}$for: (A) h = 0.1 mm; (B) h = 0.5 mm; (C) h = 1 mm; (D) h = 2 mm; (E) h = 3 mm. Datapoints are expressed as mean ± standard deviation of $\boldsymbol{v}_{\boldsymbol{pp}}$ of single action potentials fired by the neuron.


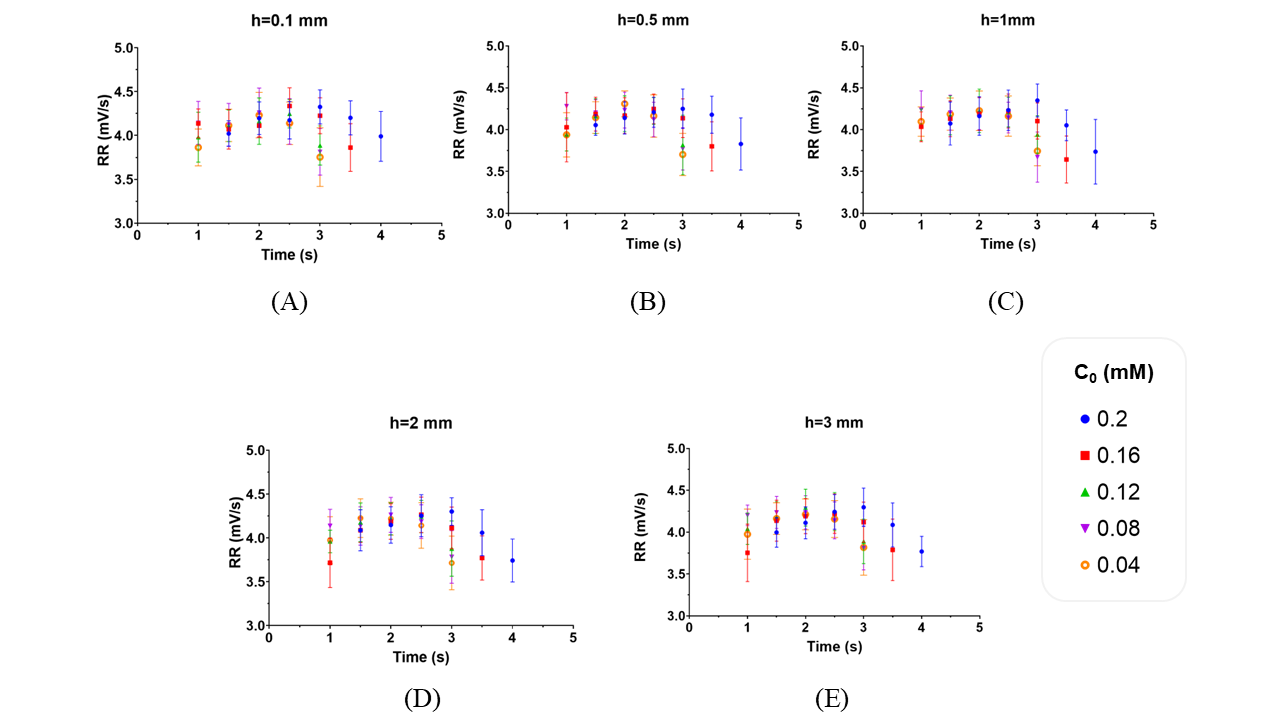


Supplementary Figure 2. Rise rate over time as a function of the oxygen concentration at the boundary $\boldsymbol{c}_{\boldsymbol{0}}$ for: (A) h = 0.1 mm; (B) h = 0.5 mm; (C) h = 1 mm; (D) h = 2 mm; (E) h = 3 mm. Datapoints are expressed as mean ± standard deviation of $\boldsymbol{rr}$ in the APs identified within a sliding window of 0.5 seconds.


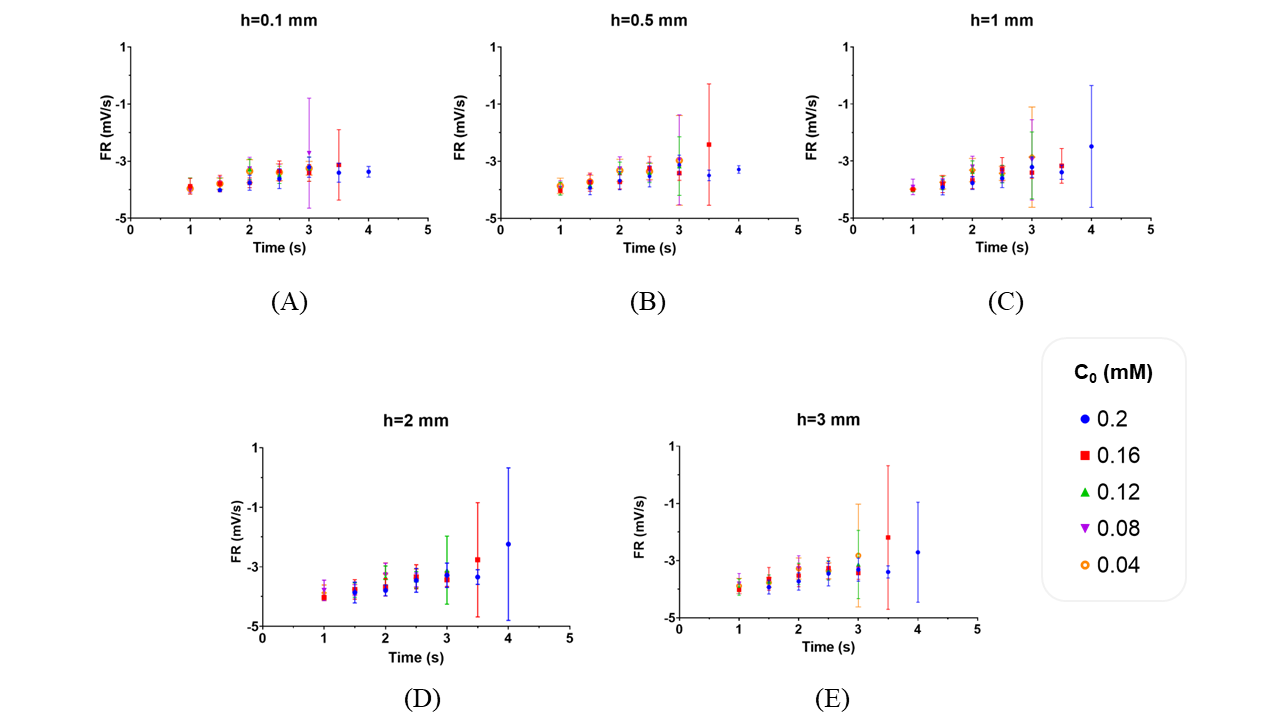


Supplementary Figure 3. Fall rate over time as a function of the oxygen concentration at the boundary $\boldsymbol{c}_{\boldsymbol{0}}$ for: (A) h = 0.1 mm; (B) h = 0.5 mm; (C) h = 1 mm; (D) h = 2 mm; (E) h = 3 mm. Datapoints are expressed as mean ± standard deviation of $\boldsymbol{fr}$ in the APs identified within a sliding window of 0.5 seconds.

Supplementary Figure 4. (A) Dissipation Rate (DR) and (B) Aspect Ratio (AR) as a function of $\boldsymbol{\Phi}^{\mathbf{2}}$. For the sake of clarity, $\boldsymbol{\Phi}^{\mathbf{2}}$ values are reported in log-scale. Datapoints correspond to the considered combinations of $\boldsymbol{c}_{\mathbf{0}}$ and $\boldsymbol{h}$ reported in Table2 of the main text. For higher $\boldsymbol{\Phi}^{\mathbf{2}}$values, the neuron fires shorter trains, which are hence characterized by lower values of $\boldsymbol{AR}$ and a faster decay of the peak amplitude (i.e., more negative values of DR). Again, a reduced sensitivity to decreasing values of $\boldsymbol{c}_{\mathbf{0}}$ can be observed at high values of $\boldsymbol{\Phi}^{\mathbf{2}}$.


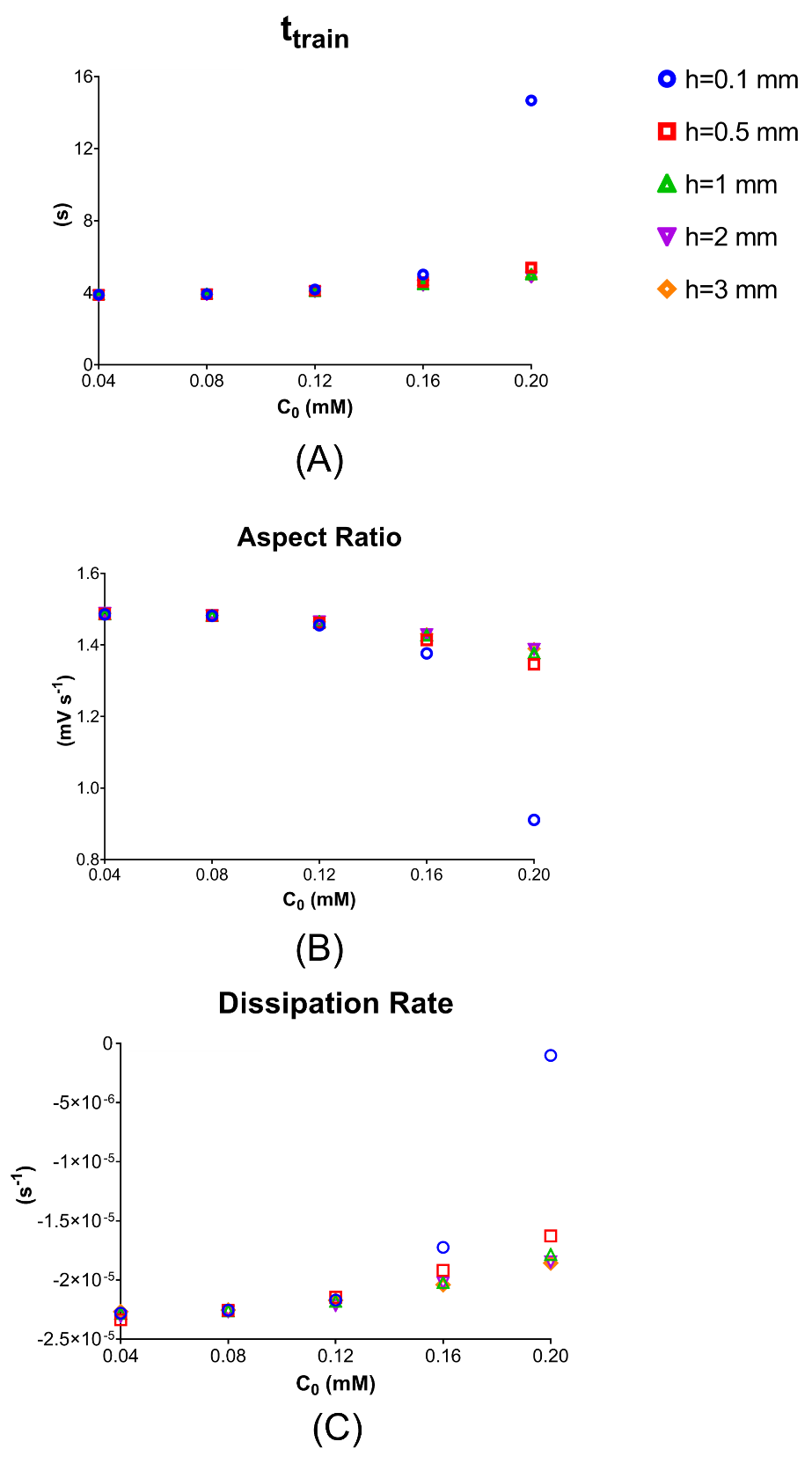


Supplementary Figure 5. (A) Time duration of the train of action potentials $\boldsymbol{t}_{\boldsymbol{train}}$, (B) Dissipation Rate and (C) Aspect Ratio varying boundary oxygen concentration $\boldsymbol{c}_{\boldsymbol{0}}$ for each of the five simulated heights of medium $\boldsymbol{h}$, 0.1, 0.5, 1, 2 and 3 mm.


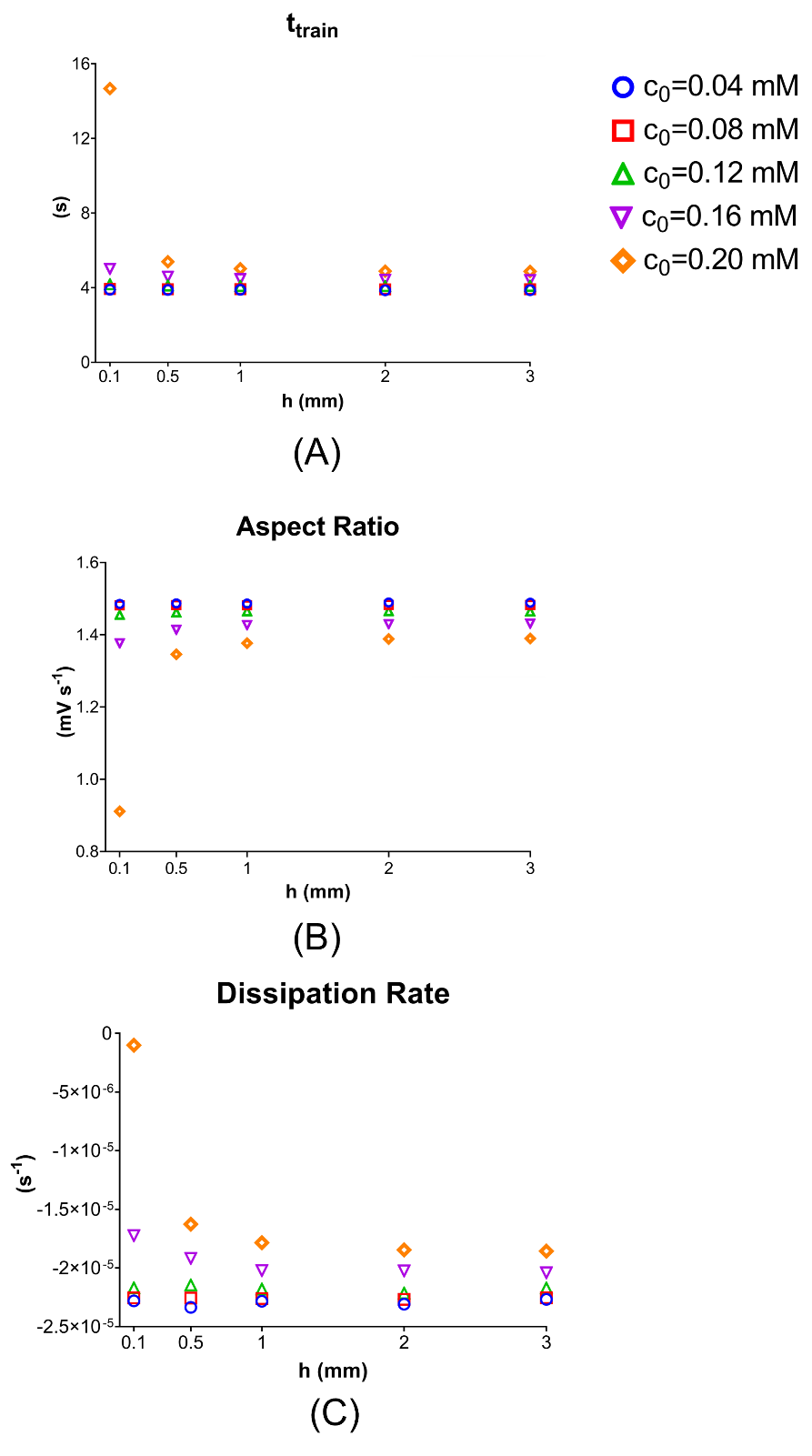


Supplementary Figure 6. (A) Time duration of the train of action potentials $\boldsymbol{t}_{\boldsymbol{train}}$, (B) Dissipation Rate and (C) Aspect Ratio varying height of medium $\boldsymbol{h}$ for each of the five simulated boundary oxygen concentrations $\boldsymbol{c}_{\boldsymbol{0}}$, 0.04, 0.08, 0.12, 0.16 and 0.2 mM.

## Supplementary Tables

Supplementary Table 1. Descriptive parameters of *in vitro* networks (Ballesteros-Esteban et al., 2023).

| **Parameter** | **Value** |
| --- | --- |
| Number of analyzed networks | 19 |
| Number of electrodes/MEA | 120 |
| Seeding cell density | 1200 cells/chip |
| Medium height ($h$) | 3 mm |
| Medium volume | 2 mL |

Supplementary Table 2. Small World metrics exploited for describing the wiring pattern of a network composed of $\boldsymbol{q}$ vertices and $\boldsymbol{s}$ links. $\boldsymbol{a}_{\boldsymbol{ij}}$ defines the connection state of vertices i and j: it is 1 if vertices i and j are connected, 0 otherwise.

| Metric | Definition | Ref |
| --- | --- | --- |
| Mean vertex degree | $K=\frac{1}{q}\sum_{i=1}^{nq} k_{i}$  $k_{i}$ is the total number of vertices $j$ to which the vertex $i$ is connected | (Rubinov & Sporns, 2010) |
| Characteristic path length | $L=\frac{1}{q}\sum_{i=1}^{q} X_{i}$  $X_{i}$ is the average distance between the $i^{th}$ vertex and all the other vertices | (Watts & Strogatz, 1998) |
| Clustering coefficient of vertex $i$ | $CC_{i}=\frac{s_{i}}{k_{i}\left( k_{i}-1 \right)}$  $s_{i}$ is the number of edges between the neighbours of vertex $i$ | (Watts & Strogatz, 1998) |
| Overall clustering coefficient | $CC=\frac{1}{q}\sum_{i=1}^{q} CC_{i}$  $CC_{i}$ is the clustering coefficient of $i^{th}$ vertex | (Watts & Strogatz, 1998) |
| Small-worldness | $\sigma=\frac{\left( \frac{CC}{CC_{rand}} \right)}{\left( \frac{L}{L_{rand}} \right)}$  $CC_{rand}$ and $L_{rand}$ refer to the values of CC and L of a random network having the same number of vertices $q$ and edges $s$ of the tested network | (Humphries & Gurney, 2008) |
| Edge density | $\xi=\frac{s}{q\left( q-1 \right)}$ | (Achard & Bullmore, 2007) |

Supplementary Table 3. Number of vertices and links of the Digitoids developed for each of the analysed DIV neuronal networks.

|  |  | **DIV 11** | **DIV 12** | **DIV 13** | **DIV 14** | **DIV 15** | **DIV 16** |
| --- | --- | --- | --- | --- | --- | --- | --- |
| ***Digitoids #1*** | # vertices | 78 | 75 | 79 | 75 | 73 | 52 |
|  | #edges | 156 | 150 | 158 | 150 | 146 | 156 |
| ***Digitoids #2*** | # vertices | 90 | 82 | 86 | 82 | 80 | 71 |
|  | #edges | 180 | 164 | 172 | 164 | 160 | 171 |
| ***Digitoids #3*** | # vertices | 105 | 88 | 92 | 89 | 90 | 89 |
|  | #edges | 210 | 176 | 184 | 178 | 180 | 178 |

**Supplementary Table 4. Values of Spearman coefficient (*r*) and associated p-values (*p*) obtained from the correlation analysis of** $\boldsymbol{t}_{\boldsymbol{train}}$ **and** $\boldsymbol{c}_{\boldsymbol{0}}$ **for the different medium heights (**$\boldsymbol{h}$**),**

| $\boldsymbol{h}$ (mm) | r | *p* |
| --- | --- | --- |
| 0.1 | 1.00 | <0.05 |
| 0.5 | 1.00 | <0.05 |
| 1 | 1.00 | <0.05 |
| 2 | 1.00 | <0.05 |
| 3 | 1.00 | <0.05 |

Supplementary Table 5. Values of Spearman coefficient (r) and associated p-values (p) obtained from the correlation analysis of AR and $\boldsymbol{c}_{\boldsymbol{0}}$ for the different medium heights ($\boldsymbol{h}$).

| $\boldsymbol{h}$ (mm) | r | p |
| --- | --- | --- |
| 0.1 | -1.00 | <0.05 |
| 0.5 | -1.00 | <0.05 |
| 1 | -1.00 | <0.05 |
| 2 | -1.00 | <0.05 |
| 3 | -1.00 | <0.05 |

Supplementary Table 6. Values of Spearman coefficient (r) and associated p-values (p) obtained from the correlation analysis of DR and $\boldsymbol{c}_{\boldsymbol{0}}$ for the different medium heights ($\boldsymbol{h}$).

| $\boldsymbol{h}$ (mm) | r | p |
| --- | --- | --- |
| 0.1 | 1.00 | <0.05 |
| 0.5 | 1.00 | <0.05 |
| 1 | 1.00 | <0.05 |
| 2 | 1.00 | <0.05 |
| 3 | 1.00 | <0.05 |

Supplementary Table 7. Values of Spearman coefficient (r) and associated p-values (p) obtained from the correlation analysis of $\boldsymbol{t}_{\boldsymbol{train}}$ and $\boldsymbol{h}$ for the different boundary oxygen concentrations $\boldsymbol{(}\boldsymbol{c}_{\boldsymbol{0}}$).

| $\boldsymbol{c}_{\boldsymbol{0}}$ (mM) | r | p |
| --- | --- | --- |
| 0.04 | -0.80 | >0.05 |
| 0.08 | -0.80 | >0.05 |
| 0.12 | -0.70 | >0.05 |
| 0.16 | -1.00 | <0.05 |
| 0.20 | -1.00 | <0.05 |

Supplementary Table 8. Values of Spearman coefficient (r) and associated p-values (p) obtained from the correlation analysis of AR and $\boldsymbol{h}$ for the different boundary oxygen concentrations ($\boldsymbol{c}_{\boldsymbol{0}}$).

| $\boldsymbol{c}_{\boldsymbol{0}}$ (mM) | r | p |
| --- | --- | --- |
| 0.04 | 0.80 | >0.05 |
| 0.08 | 0.80 | >0.05 |
| 0.12 | 0.70 | >0.05 |
| 0.16 | 1.00 | <0.05 |
| 0.20 | 1.00 | <0.05 |

Supplementary Table 9. Values of Spearman coefficient (r) and p-values (p) obtained from the correlation analysis of DR and $\boldsymbol{h}$ for the different boundary oxygen concentrations ($\boldsymbol{c}_{\boldsymbol{0}}$).

| $\boldsymbol{c}_{\boldsymbol{0}}$ (mM) | r | p |
| --- | --- | --- |
| 0.04 | 0.30 | >0.05 |
| 0.08 | 0.00 | >0.05 |
| 0.12 | -0.6 | >0.05 |
| 0.16 | -1.00 | <0.05 |
| 0.20 | -1.00 | <0.05 |

Supplementary Table 10. p-values obtained by statistically comparing (α = 0.05) the mFR output from Digitoids to that of the HH model and the experimental data at DIV from 11 to 16 (Ballesteros-Esteban et al., 2023).

| DIV | Digitoids vs Experimental | HH vs Experimental |
| --- | --- | --- |
| DIV 11 | 0.3594 | 0.0146 |
| DIV 12 | 0.0762 | 0.0049 |
| DIV 13 | 0.1551 | 0.0065 |
| DIV 14 | 0.0762 | 0.0049 |
| DIV 15 | 0.3594 | 0.0146 |
| DIV 16 | 0.1973 | 0.0077 |

# References

Achard, S., & Bullmore, E. (2007). Efficiency and cost of economical brain functional networks. *PLoS Computational Biology*, *3*(2), 0174–0183. https://doi.org/10.1371/journal.pcbi.0030017

Ballesteros-Esteban, L. M., Leyva, I., Almendral, J. A., & Sendiña-Nadal, I. (2023). Self-organization and evolution of structure and function in cultured neuronal networks. *Chaos, Solitons & Fractals*, *173*(June), 113764. https://doi.org/10.1016/j.chaos.2023.113764

Humphries, M. D., & Gurney, K. (2008). Network “small-world-ness”: A quantitative method for determining canonical network equivalence. *PLoS ONE*, *3*(4). https://doi.org/10.1371/journal.pone.0002051

Rubinov, M., & Sporns, O. (2010). Complex network measures of brain connectivity: Uses and interpretations. *NeuroImage*, *52*(3), 1059–1069. https://doi.org/10.1016/J.NEUROIMAGE.2009.10.003

Watts, D. J., & Strogatz, S. H. (1998). Collective dynamics of ‘small-world’ networks. *Nature 1998 393:6684*, *393*(6684), 440–442. https://doi.org/10.1038/30918

Wei, Y., Ullah, G., Ingram, J., & Schiff, S. J. (2014). Oxygen and seizure dynamics: II. Computational modeling. *Journal of Neurophysiology*, *112*(2), 213–223. https://doi.org/10.1152/jn.00541.2013
