# Supplementary material for: Digitoids: a novel computational platform for mimicking oxygen-dependent firing of neurons in vitro
Source: Front Neuroinform. 2025 Jul 1;19:1549916. doi: 10.3389/fninf.2025.1549916 (PMC12259620; doi:10.3389/fninf.2025.1549916)
Supplement: Supplementary file 2 [file Data_Sheet_2.zip › Digitoids_Code/Tutorial.docx]

## Repository for code of paper "*Digitoids: a novel computational platform for mimicking oxygen-dependent firing of neurons in vitro*" (Fabbri, R. et al.)

Abstract: “Computational models are valuable tools for understanding and studying biological neural networks in vitro. However, few of the current in silico approaches consider the energetic demand of neurons to sustain their electrophysiological functions, specifically their well-known oxygen-dependent firing. In this work, we introduce Digitoids, a computational platform which integrates a Hodgkin-Huxley-like model to describe the time-dependent oscillations of the neuronal membrane potential with oxygen dynamics in the culture environment. In Digitoids, neurons are connected to each other according to the Small-World topologies observed in vitro and oxygen consumption by cells is modelled as limited by diffusion through the culture medium. The oxygen consumed is used to fuel their basal metabolism and the activity of Na+-K+-ATP membrane pumps, thus it modulates neuronal firing. Our simulations show that the characteristics of neuronal firing predicted throughout the network are related to oxygen availability. In addition, the average firing rate predicted by Digitoids is statistically similar to that measured in neuronal networks in vitro, further proving the relevance of this platform. Digitoids paves the way for a new generation of in silico models of neuronal networks, establishing the oxygen dependence of electrophysiological dynamics as a fundamental requirement to improve their physiological relevance”

## Single neuron model

Download folder “Code” and unzip it to a desired location in your device. Now it will de identified by the path: local/Code.

Open MATLAB (if you do not have it already installed, visit: <https://it.mathworks.com/help/install/ug/install-products-with-internet-connection.html>, the model was developed under Matlab R2023b) and in the upper menu select “Browse for folder”
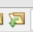
 and choose the folder where you unzipped the Digitoids code, i.e., local/Code. Now the content of the folder should appear in the “Current folder” window, usually placed on the left side of the Matlab window. Note: you should have Simulink toolbok installed to open and work with these files, if you do not have it, please add it to you Matlab.

Now, directly in the “Current folder” window double-click on the folder “SingleNeuron” and here double-click on “Single_neuron.slx’, which contains the block implementing the single neuron dynamics (as described in Section 2.1 of the Main Manusript). Once open, you see this (Figure 1):


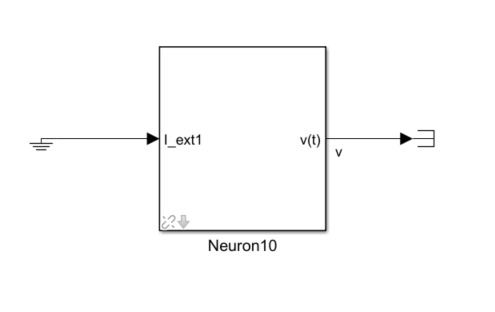


**Figure 1** Single neuron block implemented in Simulink.

Now select with your pointer the “Neuron” block and click Ctrl+U to “look under mask”, i.e., to go inside the “Neuron” block and see its components (Figure 2).


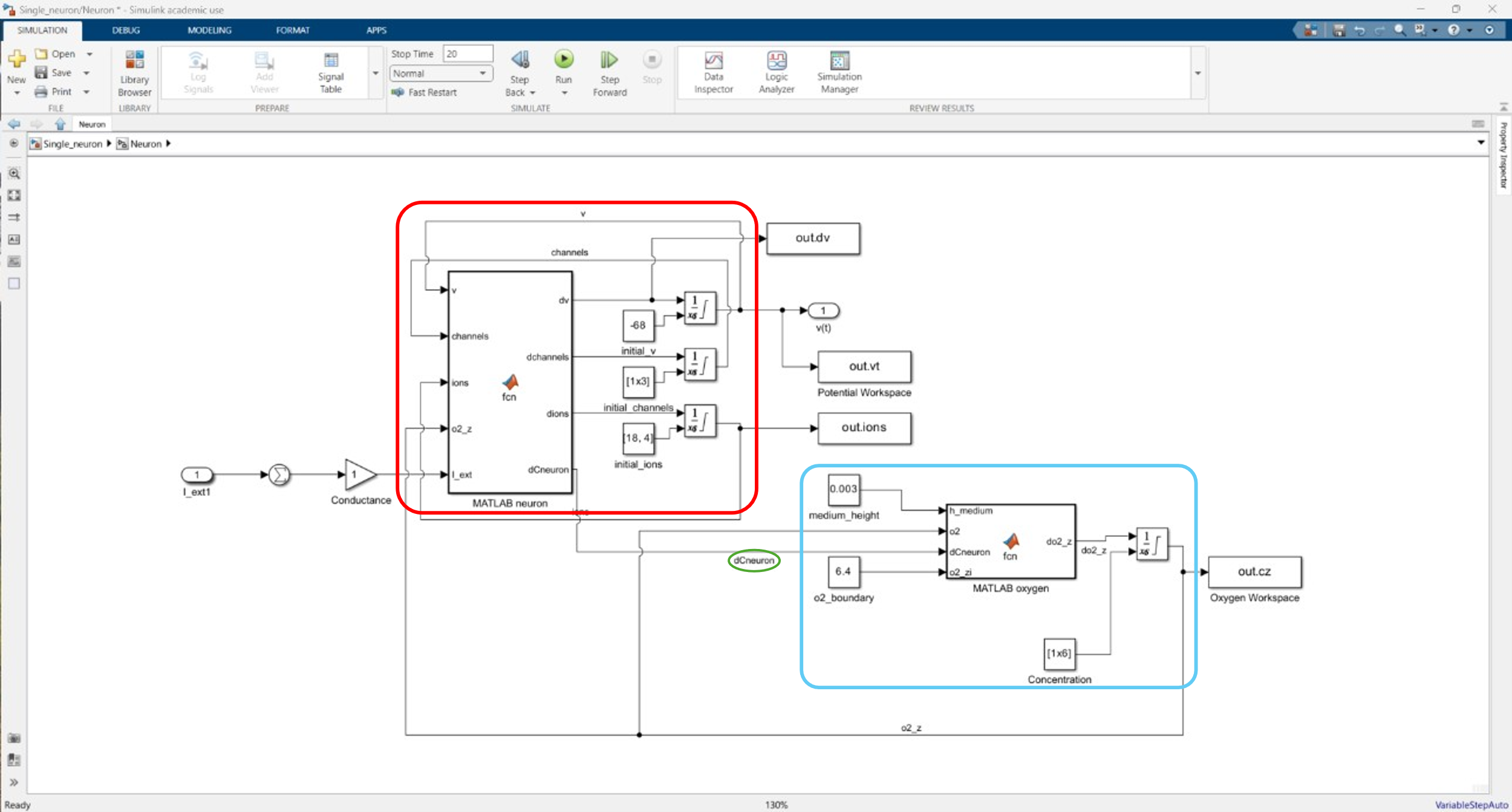


**Figure 2** Simulink scheme of the "Neuron" block, implementing single neuron dynamics where firing (red square) of neuron is coupled to oxygen diffusion (blue square) in cell culture medium through the term "dCneuron" (green circle), representing the oxygen consumed by the neuron to sustain its metabolism and functioning (as decribed in Section 2.1.1 and 2.1.2 of the Main Manuscript).

### Neuron dynamics

The block “MATLAB neuron” (red square in Figure 2) embeds the dynamics of firing and consumption regarding the single neuron, as described by Eqs. (2)-(11) in the Main Manuscript (Section 2.1.2). By double-clicking on this block, its content is opened and it is possible to see that it refers to function “neuron”. This is indeed a block that recalls a function described in an external file, in this case in the file “neuron.m”. Now, go back to Matlab window and on the “Current folder” window double-click on the file “neuron.m” to open it. At each time step $i$ of simulation, the functio “neuron.m” embedded within the “MATLAB neuron” block of the scheme calculates and outputs the following:

1. *dv* 🡪 derivative of membrane potential of neuron, calculated according to Eq. (7) of Main Manuscript (line 91 of neuron.m file);
2. *dchannels* 🡪 a (3, 1) vector collecting values of the derivatives of the activation and inactivation variables of the cell, as described by Eqs. (2)-(7);
3. *dions* 🡪 a (2,1) vector collecting the derivatives of concentrations of Na within and K outside the cell (Eqs. (11)-(12) of Supplementary Materials);
4. *dCneuron* 🡪 oxygen consumed by the cell to sustain its metabolism and functioning – i.e., for firing action potentials – described through Eq. (2) and given by the sum of two terms: one describing consumption for firing (Eq. (4) of Main Manuscript, line 102 of “neuron.m” file) and the other one describing consumption for cell metabolism (Eq. (3) of Main Manuscript and line 103 of “neuron.m” file).

The derivatives values evaluated by the function at each time step – *dv*, *dchannels* and *dions* – are then integrated through three separated “Integrator” blocks within the Simulink scheme (namely “integrator”, “Integrator 1” and “Integrator 2”), initialized with the values contained in the constant blocks “initial_v” (-68 mV), “initial_channels” ([0.9980, 0.0084, 0.5]) and “initial_ions” ([18, 4] mM).

The value output for *dCneuron* is used to realize the coupling between neuronal dynamics and oxygen diffusion ones. Indeed this values is passed as an input to the “MATLAB oxygen” block, which embeds the functions detailed in the file “oxygen_diff.m” (that you can open and inspect in the Matlab window, from the “Current folder” window (described in detail in the following Section Oxygen diffusion dynamics).

After integration, the variables to input for the “neuron” function are produced and they represent the values of the membrane potential, activation and inactivation variables, oxygen concentration in the medium at the subsequent time step $i+1$. *I_ext* is a constant, representing the eventual external applied current (in this model is set to 0, since this input of the “Neuron” block in the Simulink scheme is connected t a ground block).

### Oxygen diffusion dynamics

Oxygen diffusion dynamics through cell culture medium (height $h$) are implemented within the “MATLAB oxygen” block of the “Single_neuron.slx” Simulink model. Specifically, the file “oxygen_diff.m” implements the dynamics described through the Fick’s law, as reported in Eq. (1) of the Main Manuscript. This function calculates at each time step $i$ of simulation the derivative of oxygen concentration across the cell culture medium along the z dimension - $d02\_z$ – discretized into six steps of length $delta=h/5$. The output is then integrated into block “Integrator4” with initial value $c_{0}$, i.e., we assume that at the beginning of simulation the medium is equally oxygenated and the concentration is the same as the one at the boundary with air, i.e., $c_{0}$. The integrated value output from “integrator4” - $o2\_z$ – is the input for both the “MATLAB neuron” and “MATLAB oxygen” blocks for following simulation time step $i+1$. The “oxygen_diff” function also takes as input the oxygen consumed by the neuron $dCneuron$ which is used as a time-varying boundary term for solving the differential equations describing oxygen diffusion through the medium, thus acting as a sink at the bottom of the cell culture plate and triggering oxygen diffusion from the top to the bottom of the medium.

In this way the coupling between oxygen diffusion dynamics within the culture medium and neuron firing and metabolism dynamics are coupled, as described in Section 2.1 of Main Manuscript.

### Single neuron simulations

To run the simulations described in Section 2.2 of Main Manuscript where the values of $c_{0}$ and $h$ are varied across different configurations (Table 2), open the script contained in the file “run_sims_varying_h_c.m”. Firstly, create a new folder “Results” within the current folder “SingleNeuron”. You can do this by right clicking on the “Current Folder” window and select New >> Folder and write the name “Results”. After doing that, you can click within the script “run_sims_varying_h_c” and, in the top panel, click on the button “Run” in the “Editor” menu. This command runs the script and launch simulations of the “Single_neuron.slx” scheme varying the values of $c_{0}$ and $h$ across the values described in lines 2 and 3. The output of each simulation is saved in the folder Results and the file is named “out_h_value of h_c_value of c.mat” where the “value of h” and “value of c” correspond to the specific configurations of parameters simulated in that model.

To obtain the trends of $v(t)$ and $c(0, t)$ depicted in Figure 4, you can launch the script “multiple_plot_vc.m” where the output of each single-neuron-simulation is used to extract time, trend of membrane potential and of oxygen concentration at $z=0$. By changing the value of $h$ in line 2 (*srcfile*=…) it is possible to select the output of simulation of different values of $h$.

Then, you can calculate the metrics relative to the train of action potentials (described in Section 2.3 of Main Manuscript) by launching the script “train_metrics_extraction.m” where $t_{train}$, AR and DR are assessed for each output of the simulations performed by varying $h$ and $c_{0}$, as well as the value of $\Phi^{2}$, as described by Eq. (21) in Main Manuscript. These values are then saved in a structure within the folder “Results” as “metrics_res.mat”. The values calculated from the simulations described in the Main Manuscript are stored in the variable saved as “metrics_res.mat” within the “SingleNeuron” folder.

The single action potential metrics are calculated through the “AP_metrics_extrcation.m” file. By running this script another structure – *res_ap* - is created where single action potential metrics are stored and saved as “metrics_res_ap.mat” file.

By running the script “generate_scatterplot_ttrain_AR_DR.m” the plot depicted in Figure 5.B of Main Manuscript and Supplementary Figures 4.A and 4.B can be created. Indeed, the values of $t_{train}$, AR and DR ad a function of $\Phi^{2}$ are scattered in these Figures.

The file “ttrain_AR_DR.xlsx” contains the values of $t_{train}$, AR and DR organized into six different sheets. This file provide the tables that can be used to perform the non-linear correlation analysis described in Section 2.3 of Main Manuscript. Results presented in Section 3.1 were obtained exploiting GraphPad Prism, where the tables in the above-mentioned file were imported and analyzed to assess Spearman coefficient.

## Digitoids models

In the window “Current Folder” you can go back to the “Code” folder. This folder contains the following files:

1. 'adapthr_spikedetection.m': function for detecting spikes with the adaptive threshold;
2. 'define_Digitoids.m': starting from the variables stored in file “Models_data.m” this script creates the Simulink scheme of the correspondent Digitoids positioning the neuron blocks and wiring them as described in Section 2.1.3 and 2.1.4 of Main Manuscript, according the the parameters of “Models_data”;
3. 'define_HHmodels.m': starting from “Models_data.m” this script creates the correspondent Hodgkin-Huxley models – thus, not implementing the oxygen-dependent dynamics of Digitoids - positioning the neuron blocks and wiring them with the same pipeline adopted for creating Digitoids;
4. 'metrics_analysis.m': suggested pipeline for analysing the output of the simulated Digitoids and Hodgkin-Huxley models where firstly data output from network simulations are imported, threshold for spike detection is defined, the single neuron output of each cell within the network is analyzed and mean firing rate is assessed;
5. 'Models_data.mat': the data of the experimental network layout identified in the paper "Ballesteros-Esteban, et al., 2023" and processed with the Watts-Strogatz function by MATLAB (the user can define different network layouts using the same metrics defined in “Models_data”).
6. 'neuron.m': MATLAB function called within the single neuron block in the Simulink “Digitoids_lib” library. It defines the oxygen-dependent model of firing and metabolism, as described in the Main Manuscript.
7. 'Digitoids_lib.slx': this is the Simulink library where the single neuron blocks are defined. Each user can tune and define further blocks or modify the existing to tailor the desired characteristics.
8. 'neuron_nodiff': Hodgkin-Huxley model with no dependence on energetic considerations
9. 'oxygen_diff.m': MATLAB function called within the single neuron block in the Simulink “Digitoids_lib” library. It defines the diffusion of oxygen across the column of medium above the neuron and its triggering by the single neuron consumption, as described in the paper.
10. 'sl_customization.m': function to be called before using the Neuron library to see it appear at the top of the Simulink Library browser
11. 'slblocks.m': function to be called before using the Neuron Library to see it appear with the desired name in the Simulink Library browser

Firstly, by running the script “define_Digitoids” it is possible to build Digitoids with Small-World layout with number of vertices and edges as observed in the cultured neuronal networks by Ballesteros-Esteban et al, 2023. Here, the parameters stored in “Models_data” are loaded. The name of the Simulink model – i.e., of the Digitoid you are building – can be defined at line 7 of the script. At line 27, the variable *initial_concentration* holds the value to be given to $c_{0}$ (in mg/l), i.e, the boundary oxygen concentration in the developed network model. At line 28, variable *medium_height* sets the value to be given to the medium height $h$ (in m). Default values are 6.4 mg/l for $c_{0}$ and 3 mm for $h$, being the most common values in cell cultures. Then, the network layout is defined: a “Neuron” block from the “Digitoids_lib” is placed at every vertex of the Small-World graph defined through the parameters of “Models_data”, inputting it with the user-defined parameters (i.e., $c_{0}$, $h$, name of the block in the model, number of inputs it receives from other neurons). The wiring is obtained by connecting the neurons within the Digitoid under development according to the edges defined in “Models_data”. When a neuron is not connected to others, its inputs are connected to a “Ground” block or its ouputs to a “Terminator” one. Then each system is saved with the defined model name, as selected by the user.

At this time, by opening the Digitoids saved within the folder “Digitoids_DIV11_DIV16”, i.e., the Digitoids developed exploiting the *in vitro* data as described in Section 2.1.4 of Main Manuscript, they can be run for a user-defined time interval and the output is produced in the Matlab workspace as a variable “out” where the values of time (“out.tout”), oxygen concentration (“out.cz1.signal.values” for Neuron 1 in the Digitoid) and membrane potential (“out.vt44.signals.values” for Neuron 44 in the Digitoid) are stored for each cells constituting the simulated Digitoid network. Manually save the variable “out” by right-clicking on it directly in the “Workspace” Matlab window and selecting “Save as”. Then, save it in a desired folder. You can use it for further analyses, described in the script “metrics_analysis”, by changing folder name and file name according to the ones chosen by the user.


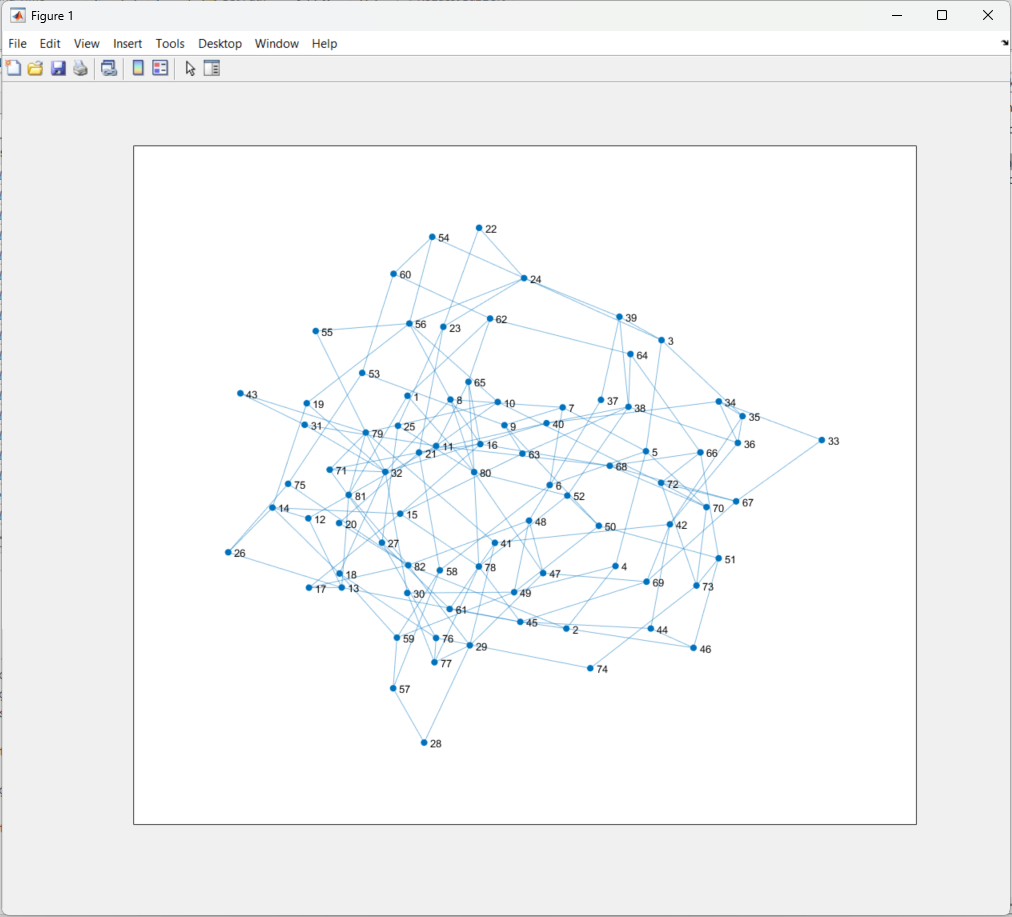

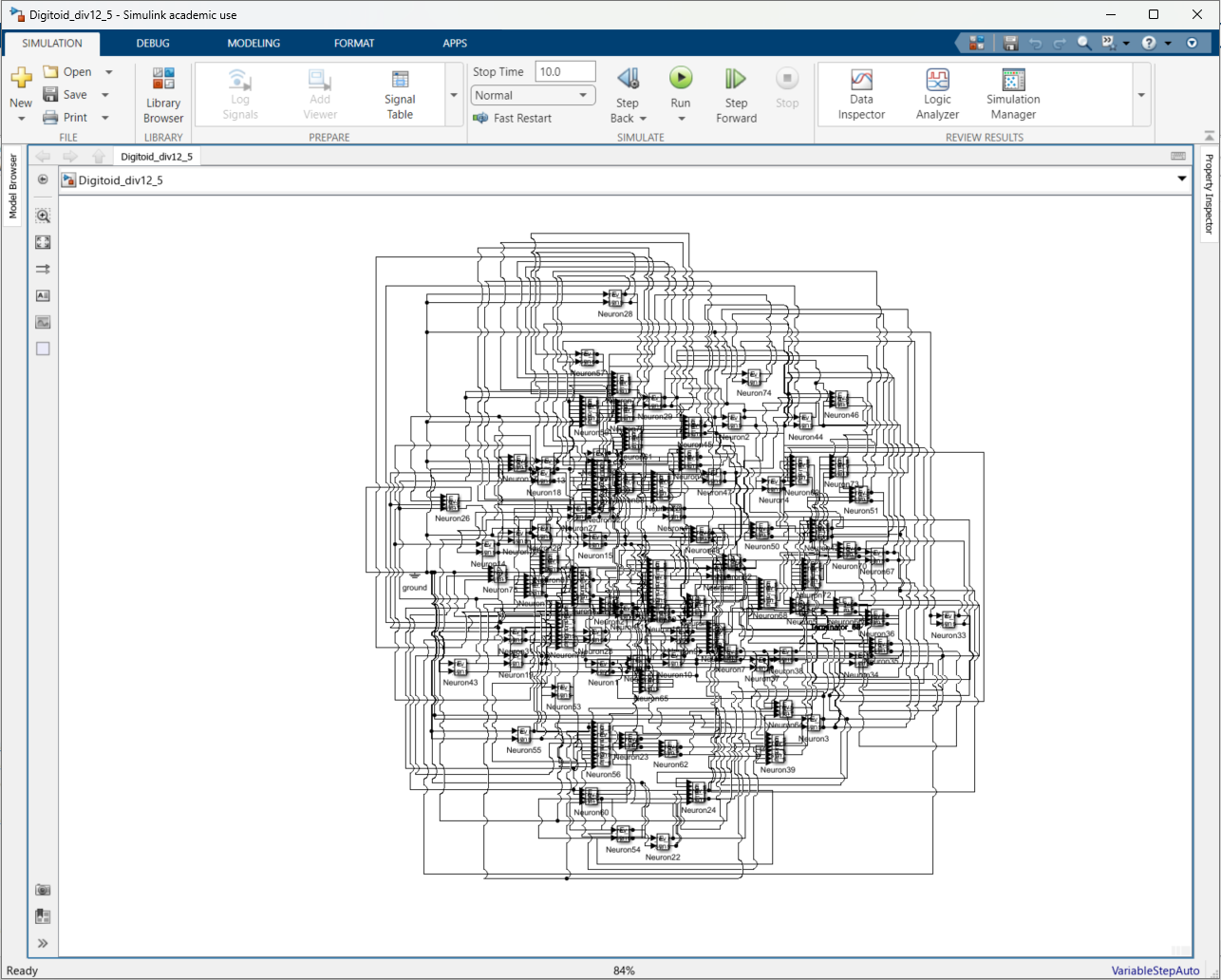


**Figure 3** Example of the rationale adopted to build Digitoids: on the left side of the Figure there is the graph obtained from the Watts-Strogatz function with the number fvertices and edges as the one calculated in vitro in Ballesteros-Esteban et al., 2023; on the right side there is the correspondent Digitoids, obtained by placing a neuron block in every vertex position and wiring the neurons with the same adjacency matrix as the one of the graph.

The same procedure described above to build Digitoids can be adopted to build HH networks. This is realized by launching the script “define_HHmodels.m” where the blocks defined within the library with non-oxygen-dependent dynamics are exploited. Then, the networks can be run and the produced output stored in the variable “out” can be saved to a defined folder and used for following analyses.
